# Comparison Between Ultrasonic and Thermal Exfoliation for Molybdenum Disulfide Nanoparticles—A Multifunctional Study

**DOI:** 10.3390/nano16171058

**Published:** 2026-08-25

**Authors:** Hon Pan Yiu, William Li, Jang-Hsing Hsieh, Chuan Li, Cho Yin Lee, Raman Sankar

**Affiliations:** 1 Department of Biomedical Engineering, National Yang-Ming Chiao Tung University, Taipei 112304, Taiwanli.wil@northeastern.edu (W.L.); jhhsieh@mail.mcut.edu.tw (J.-H.H.); 2 Department of Radiation Oncology, Taoyuan General Hospital, Ministry of Health and Welfare, Taoyuan City 330, Taiwan; 3 Institute of Physics, Academia Sinica, Taipei 115201, Taiwan; sankarraman@gate.sinica.edu.tw

**Keywords:** molybdenum disulfide, ultrasonication, solvent–thermal exfoliation, photoluminescence, photothermal effects

## Abstract

The transition metal dichalcogenides (MX_2_, TMD) are a family of two-dimensional compounds, and MoS_2_ is perhaps the most representative material among them. MoS_2_, in its bulk form, is semiconductive, with an indirect band gap of around 1.2–1.3 eV. Monolayer MoS_2_, nevertheless, exhibits a direct band gap of 1.8–1.9 eV. For zero-dimensional MoS_2_ (quantum dots), the band gap would be even wider. These changes are mainly due to quantized energy levels arising from geometric confinement. Quantum effects directly yield distinct optoelectronic properties, such as photoluminescence, which are absent in the material’s bulk form. In this study, we developed an economical, scalable one-pot synthesis of MoS_2_ nanoparticles at atmospheric pressure, eliminating the need for high-pressure autoclaves commonly employed in hydrothermal synthesis at around 150–270 °C. This one-pot synthesis was tested by two exfoliation methods—ultrasonication and thermal heating at 80 °C in three solvents: ethanol, deionized water, and N-methyl-2-pyrrolidone. Our focus is on the empirical study of nanoparticles’ photoluminescence and photothermal effects. The material characterization includes transmission electron microscopy for morphology and crystal structure, X-ray photoelectron spectroscopy for chemical bonding energies, and a UV–visible–NIR spectrometer for optical absorption. Photoluminescent spectroscopy of the nanoparticles was assessed using laser excitation between 300 and 400 nm, and photothermal effects were measured as temperature variations in DI water under continuous irradiation by an 808 nm laser. Results show that MoS_2_ nanoparticles synthesized in ethanol exhibit an additional broad optical absorption in the red–infrared range, in addition to the usual high absorption in the UV–blue range. This photothermal absorption would raise the temperature of DI water to 50 °C in 300 s. A simplified numerical analysis by finite elements was also carried out as a numerical confirmation of the photothermal test. On the cell biological side, both in vitro cultures with NIH/3T3 fibroblasts and antibacterial against *E. coli* show that critical tolerance of MoS_2_ is no more than 0.25 mg/mL in the media.

## 1. Introduction

Molybdenum disulfide (MoS_2_) belongs to the class of transition metal dichalcogenides with a silvery black appearance [1,2]. Bulk MoS_2_ is a semiconducting material with an indirect band gap of approximately 1.2 eV. It has been considered for photovoltaic and photocatalytic applications due to its strong absorption in the solar spectrum [3,4,5,6]. When the bulk size is reduced to a two-dimensional layer with a thickness of around 0.65–0.75 nanometers [7,8], geometric confinement would make quantum effects (discrete energy levels) more pronounced, thereby significantly affecting the optoelectronic properties of MoS_2_. More specifically, the widened band gap in two-dimensional layers leads to higher photon–electron exchange energies and greater potential for electron-hole separation [9,10,11]. One corresponding phenomenon of these changes is photoluminescence, which has been observed in MoS_2_ films, nanoplates, and nanotubes [12,13,14].

The zero-dimensional MoS_2_ nanoparticles remain semiconductive, but quantum effects are expected to be even more pronounced [15,16,17]. However, changes in band structures could further induce additional optical absorption in the near-infrared range. This absorption can also excite electrons and initiate photothermal processes, in which excited electrons transfer their energy to the lattice, creating additional lattice vibrations (phonons). Phonons usually release energy non-radiatively, for example, as heat. Heat can enhance materials’ chemical (e.g., catalytic) activities or other physical effects (diffusion, thermal deformation, phase transitions, etc.) [18,19,20].

The photoelectronic interactions and photothermal effects in nanoparticles have numerous applications. For example, energy is harvested in solar cells [21,22], optical sensing and imaging [23,24], spectroscopy [25], industrial heating [26,27], photocatalytic reactions [28], antibacterial applications [29,30,31], and photothermal diagnosis/therapy for cancer [31,32,33]. In this study, we focus on evaluating the photocatalytic and photothermal effects of synthesized MoS_2_ nanoparticles.

There are two categories of MoS_2_ nanoparticle production. Both top-down and bottom-up approaches are presently available. For example, mechanical and chemical exfoliation, electrochemical lysis, emulsion, solvent–thermal, thermal ablation, and combined methods have been studied [13,34,35]. A common problem across all these approaches is low production yields and the need for relatively high temperatures (150–270 °C) in autoclaves [13,35,36]. Low yield can be affected by many factors, such as solvent concentration, temperature, processing time, and the power of external sources (electrical or mechanical) [14,37,38].

Besides production, collecting nanoparticles with sufficiently fine filters can hinder upscaling production [37,38,39]. Storing nanoparticles in colloids or suspension form is a common practice [40]. But this storage could limit their applications in solid-state devices. Note that MoS_2_ nanoparticles are much heavier than their counterparts, carbon QDs, so upscaling industrial production is more challenging [40,41].

The synthesis of MoS_2_ nanoparticles in solvents is relatively simple and environmentally friendly [42,43,44]. MoS_2_ powder is first dispersed in solvents such as N-Methyl-2-pyrrolidone (NMP), ethanol (EtOH), and deionized water (DIW) [45]. Then, using either ultrasonic or thermal exfoliation, the MoS_2_ nanoparticles can be produced.

The selection of these three solvents is based on their relatively high solubility of MoS_2_ (ethanol: 1–5 mg/mL; NMP: 20–40 mg/mL) [46,47,48,49,50], and DI water (0.14–0.8 g/mL) [51,52,53] is mainly used as a base for comparison. Note that solubility is not a simple physical property and involves a variety of other molecular/surface properties, such as polarity or surface tension. From the fabrication viewpoint, however, solubility is a key factor at the outset of the whole process.

In this study, we developed a cost-effective, one-pot synthesis for MoS_2_ nanoparticles under atmospheric pressure. Two methods were used: ultrasonication and thermal exfoliation at 80 °C to synthesize MoS_2_ nanoparticles in the three solvents mentioned above. For material characterization, the particle size and morphology of these particles were examined by transmission electron microscopy (TEM); the chemical bonding energy of Mo and S was analyzed by X-ray photoelectron spectroscopy (XPS), and optical absorption and photoluminescence (PL) were detected by UV–visible–IR and PL spectrometer. These two tests also provide measures for the band gaps and optoelectronic energy conversion under irradiation. The photothermal effects of these particles was measured by a customized photothermal system. Lastly, the biotoxicity of nanoparticles in culture media was assessed by an in vitro cell culture of NIH/3T3 fibroblast cells for potential clinical thermal therapies. In addition, the chemical potency of nanoparticles can be antibacterial; therefore, an in vitro test against *E. coli* was investigated. These results could inform further research into the more efficient manufacture of MoS_2_ nanoparticles with multipurpose functions.

## 2. Materials and Methods

### 2.1. Synthesis

Commercially purchased MoS_2_ powders (approximately 100–150 μm, purity > 98%, ACROS Organics, Pittsburgh, PA, USA) were dispersed in solvents by either ice-bath ultrasonic exfoliation (UE) or solvent–thermal exfoliation (TE) for further processing.

Three solvents were chosen in this study based on their solubility and dispersibility: ethanol (EtOH, 99.99%, ECHO Chemical Co., LTD., Toufen City, Taiwan), deionized water (DIW), and N-methyl-2-pyrrolidone (NMP, ≤99.7%, Fisher Scientific, Loughborough, UK) [45]. Note that dispersibility is mainly due to the weaker intermolecular forces of liquids, such as hydrogen bonds or van der Waals forces, which allow solid particles to be accommodated or move freely to form a suspension if particles are sufficiently small.

The fabrication process is stated as follows. (1) A volume of 20 mL of each solvent was used to disperse MoS_2_ powders (200 mg) via either ultrasonic exfoliation (UE) or thermal exfoliation (TE, 16 h). (2) For ultrasonic exfoliation, the MoS_2_ mixed solution was treated by a sonicator (UC-08A, 40 kHz, 60 W, BIOBASE, Jinan, Shandong, China) for 16 h. (3) For thermal exfoliation, the MoS_2_ mixed solution was heated in a nitrogen environment (500 mL/min, around 80 °C) for 16 h with a system of a heat source (550 W) and a condenser.

After exfoliation, we carried out a two-step purification process. First, the solutions were thoroughly mixed with ethanol (95% purity) or deionized (DI) water sequentially in a volumetric ratio of 2:3, then centrifuged at 10,000 rpm for 15 min separately to remove the solvent and large-sized MoS_2_ particles. This liquid-extraction process was repeated twice.

After liquid extraction, the supernatant was abstracted and heated at 50 °C for at least 24 h. This low-temperature drying allows evaporation of the volatile solvent but avoids oxidation of the samples.

After the two-step purification, the final product was resuspended in water. Table 1 shows the experimental design of solvents and the dispersion method.

### 2.2. Transmission Electron Microscopy (TEM)

The images and morphology of MoS_2_ nanoparticles were examined by transmission electron microscopy (TEM, JEM-2000EX II, JEOL, Tokyo, Japan). The operation was conducted at an accelerating voltage of 60 kV. Samples for TEM studies were prepared by drop-casting onto standard lacy carbon TEM (Cu) grids (01800-F, PELCO^®^, Ted Pella Inc., Redding, CA, USA). The particle size was analyzed by ImageJ^®^ 1.54i in TEM images (*n* ≥ 30).

### 2.3. X-Ray Diffraction (XRD)

The crystal structure of MoS_2_ nanoparticles was analyzed by an X-ray diffraction meter (XRD; PANalytical X’Pert Pro, Malvern Panalytical, Malvern, Worcestershire, UK) with a Cu-Kα radiation source (λ = 1.54425 Å) at an incident angle of 0.5°. The scanning range (2*θ*) was from 10° to 90°, with a step size of 0.06° and 2 s per step.

The crystal size (*d*) of MoS_2_ nanoparticles can be calculated using Scherrer’s formula, i.e.,

(1) $$d=\frac{k\lambda }{\beta \cos\theta },$$

where *k* is the shape factor (0.9), *λ* is the wavelength of X-rays (1.5425 Å), *θ* is the scattering angle of the crystal plane, and *β* is the full width at half maximum (FWHM) of the peak. The numerical analysis was carried out using Highscore Plus (Version 3.0.5, Malvern Panalytical, Malvern, Worcestershire, UK) using the Gaussian function. Relevant PDF cards for MoS_2_ from the database are PDF#03-065-0160 (PDF-2 2004, The International Center for Diffraction Data, Newtown Square, PA, USA).

### 2.4. Zeta Potential

The dispersion stability of MoS_2_ nanoparticles in different solvents is measured by zeta potential (Zetasizer Nano, ZS90, Malvern, Worcestershire, UK) to assess the magnitude of electrostatic interactions (repulsion or attraction) between charged particles. This measure indicates the difference in electrical potential between the surface of a solid particle and the surrounding liquid (the slipping plane). Usually, a higher absolute zeta potential (around 30 mV) indicates stronger electrostatic repulsion between particles, resulting in a stable dispersion by inhibiting particle aggregation.

### 2.5. X-Ray Photoelectron Spectroscopy (XPS)

The chemical binding energy of synthesized MoS_2_ nanoparticles was examined by an X-ray photoelectron spectrometer (XPS, PHI 5000 VersaProbe III, ULVAC-PHI Inc., Chigasaki, Kanagawa, Japan). The energy of the X-ray beam can cause electrons from 1 to 10 nm underneath the surface to escape, which gives a direct measurement of the binding energy of the emitted electrons in the following expression:

(2) $$E_{binding}=h\nu -(E_{K.E.}+\varphi )$$

where *E*_binding_ is the binding energy of electrons, *hν* is the energy of the X-ray, *E*_K.E._ is the kinetic energy of the electron, and *φ* is the work function of the nanoparticle’s surface. The binding energy can be used to determine the types of chemical bonds and, thus, the material’s composition. The system operating conditions were set by the manufacturer as follows: chamber vacuumed to 6.7 × 10^−8^ Pa, 24.5 W power, 15 kV voltage, step size of 0.1 eV, and pass energy of 55 eV. The spectrum was numerically deconvoluted using a Gaussian function in Fityk 1.3.1.

### 2.6. UV–Visible–NIR Spectroscopy

The optical absorption of the MoS_2_ suspension was measured using UV–visible–NIR spectroscopy (Biochrom Ultrospec 9000pc, Fisher Scientific, Arendalsvägen, Göteborg, Sweden). The optical absorption range was set to 300–800 nm. The absorption was evaluated based on the Beer–Lambert law:

(3) $$I_{T}=I_{0}e^{-\varepsilon lc}$$

where *I_T_* and *I*_0_ are the intensities of the transmitted and incident laser light, ε is the molar attenuation coefficient (absorptivity) of the attenuating species, *l* is the optical path length, and *c* is the concentration of the attenuating species. The optical absorption (*A*) is defined as

(4) $$A=\varepsilon lc=-\ln\left(\frac{I_{T}}{I_{0}}\right)=-\left(ln10\right){log}_{10}(\frac{I_{T}}{I_{0}})$$

where log_10_ is used experimentally for *A* instead of the natural log; note that the attenuation of incident light is assumed to be absorption only in the homogeneous solution (MoS_2_ nanoparticle suspension).

### 2.7. Photoluminescence (PL)

The photoluminescence (PL) spectrum of MoS_2_ nanoparticles was measured with a multimode microplate reader (SPARK^®^, TECAN, Männedorf, Switzerland). The excitation laser light source ranged from 300 nm to 400 nm, with a resolution of 10 nm. To avoid overlap between the excitation and emission signals, a 45 nm blue shift in the emission was preset and recorded by the system.

### 2.8. Photothermal Test

**Figure 1:**
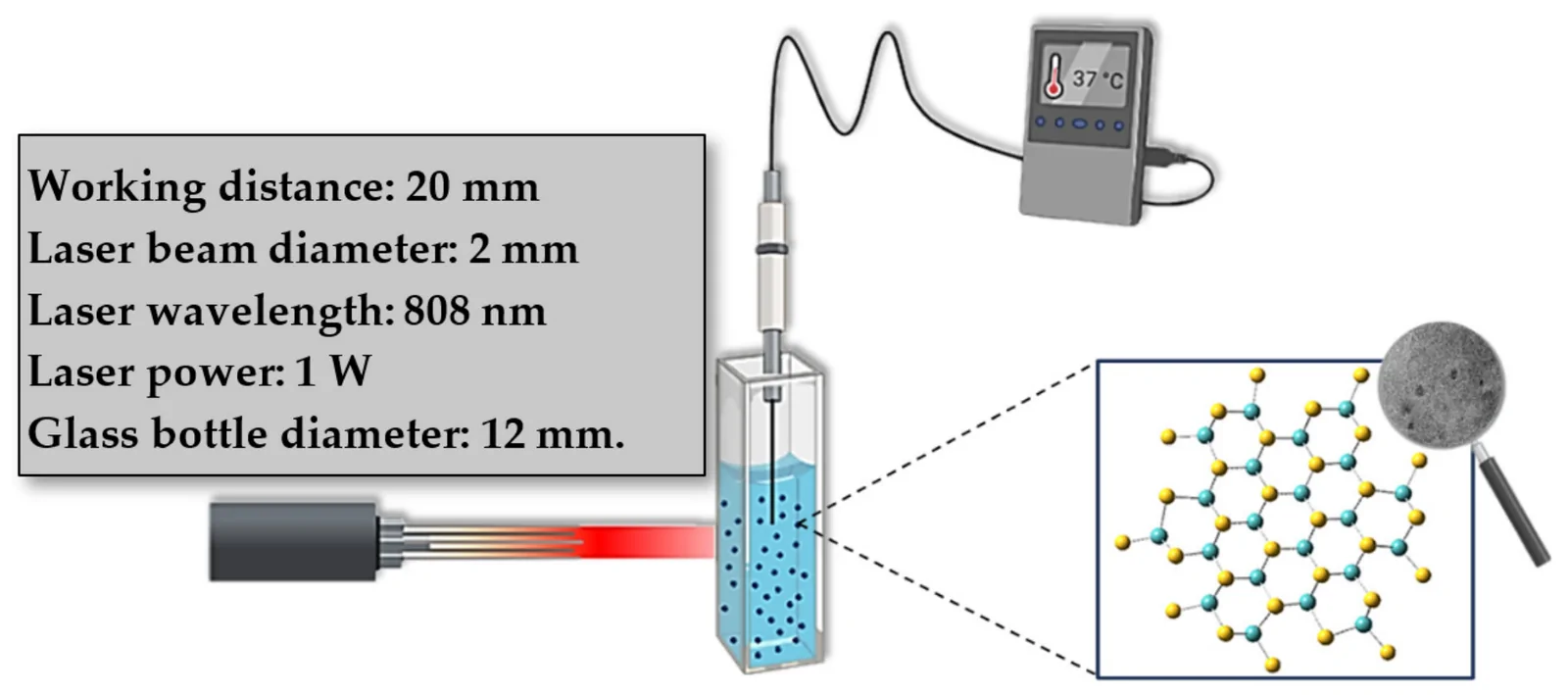
Schematic representation of the setup for the photothermal test of MoS_2_ nanoparticles in DI water.

The setup for the photothermal measurement is schematically shown in Figure 1, where an 808 nm near-infrared (NIR) light-emitting diode (LED) laser (KO-LD 808 nm 1 W, Koodyz Technology Co., Ltd., New Taipei City, Taiwan) directly irradiates MoS_2_ nanoparticles in DI water at a distance of 20 mm. The sample was stored in a glass bottle, and the test was conducted in a 3D-printed black enclosure to minimize external optical interference. The temperature was recorded by a digital thermometer (600 V CAT III, Fluke Corporation, Everett, WA, USA) every 15 s. The specific near-infrared laser (808 nm) was selected for its high transmission through human skin and its broad range of therapeutic benefits, including reduced inflammation, pain relief, increased local metabolite release, and increased blood flow [54,55,56,57,58,59].

### 2.9. In Vitro Biocompatibility

NIH/3T3 fibroblast cells (CRL-1658 ™, American Type Culture Collection, ATCC, Manassas, VA, USA) were chosen to test the toxicity of MoS_2_ nanoparticles in culture media. Cells were seeded in a 96-well tissue-culture plate at a density of 1 × 10^4^ cells per well in a culture medium (Dulbecco’s Modified Eagle Medium (DMEM), Tibco, Palo Alto, CA, USA). The incubator (Thermo Forma 310, Thermo Fisher Scientific, Waltham, MA, USA) was set to 37 °C, 95% humidity, and a 5% CO2 flow. Before cell culture, MoS_2_ nanoparticles were sterilized by UV light for 30 min to avoid contamination.

Five different concentrations of MoS_2_ nanoparticles (5 mg/mL in phosphate-buffered saline (PBS); final concentrations: 0, 0.5, 0.25, 0.125, and 0.0625 mg/mL) were added to the culture medium to test the in vitro toxicity. Note that PBS in the control group (0 mg/mL) is used to account for the solvent effects in the culture medium. Cell viability was assessed at 24 and 72 h after seeding using a 3-(4,5-dimethylthiazol-2-yl)-2,5-diphenyltetrazolium bromide (MTT) assay. The MTT assay was measured using a multimode microplate reader (SPARK^®^, TECAN, Switzerland) at 560 nm. The viability of NIH/3T3 fibroblast cells was calculated (*n* = 6) based upon the untreated (zero MoS_2_ nanoparticles, the control group) cells as follows:

(5) $$Relative\;Cell\;viability\left(\%\right)=\frac{Average\;of\;Intensity\;of\;Testing\;Group}{Average\;of\;Intensity\;of\;Control\;Group}$$

### 2.10. Antibacterial Test

The antibacterial assay followed ASTM E2149-20. *Escherichia coli* (*E. coli*, ATCC 11303™, American Type Culture Collection, ATCC, VA, USA) was used to test the antibacterial activity of MoS_2_ nanoparticles in nutrient broth (DIFCO™ Nutrient broth, Becton, Dickinson and Company, Franklin Lakes, NJ, USA). The concentrations of MoS_2_ nanoparticles were set at 0, 0.25, 0.125, and 0.0625 mg/mL for the test. Two extra groups were added for comparison. The control (negative) group is the culture with *E. coli* only, and the positive control is the one with penicillin–streptomycin solution (100 IU/mL, Biorion Inc., New Taipei City, Taiwan).

All MoS_2_ nanoparticle suspensions were irradiated by UV light for 30 min to avoid contamination before culture.

Active bacteria were first diluted in sterile nutrient broth (3 g beef extract and 5 g peptone) to an optical absorbance of 0.30 at 475 nm (~1.5–3.0 × 10^8^ CFU/mL), which served as the seed. Then, this seed was further diluted 100-fold in sterile nutrient broth before culture. During culture, a multimode microplate reader (SPARK^®^, TECAN, Switzerland) was used to assess the density of *E. coli* by measuring the medium’s optical absorbance at 600 nm (OD600).

After a 24 h incubation period, the colony-forming unit (CFU) method was used to quantify *E. coli* activity. The CFU method involves (i) a series of dilutions to ensure isolated, countable colonies on plates and (ii) standard agar plates to carry spread dilutions, as well as incubation for 16 h at 37 °C.

The colony count uses the following formula:

(6) $$CFU/mL=\frac{number\;of\;colonies}{Volume\;of\;Plate\;(mL)\;\times \;dilution\;factor}$$

The dilution factor is the ratio of the final volume to the initial volume of the aliquot during a dilution.

### 2.11. Numerical Simulation

The numerical simulation for the photothermal test was carried out by the finite element method using Elmer version 9.0 [60]. Details of the geometric model, boundary conditions, and numerical algorithms are discussed later in the Supplementary Materials.

## 3. Results

### 3.1. Yield of Synthesis

The synthesis yields of MoS_2_ nanoparticles achieved by different methods are presented in Table 2. Note that the MoS_2_ weight is based on its dry mass. Ethanol (EtOH) yielded the highest yield of 15% among the three solvents, particularly in the solvent–thermal exfoliation (TE). This can be attributed to the high solubility of MoS_2_ in ethanol (approximately 0.1–5 mg/mL) compared to the other two solvents, which enables a higher exfoliation rate [50].

### 3.2. Characterization

#### 3.2.1. Morphology and Particle Size

**Figure 2:**
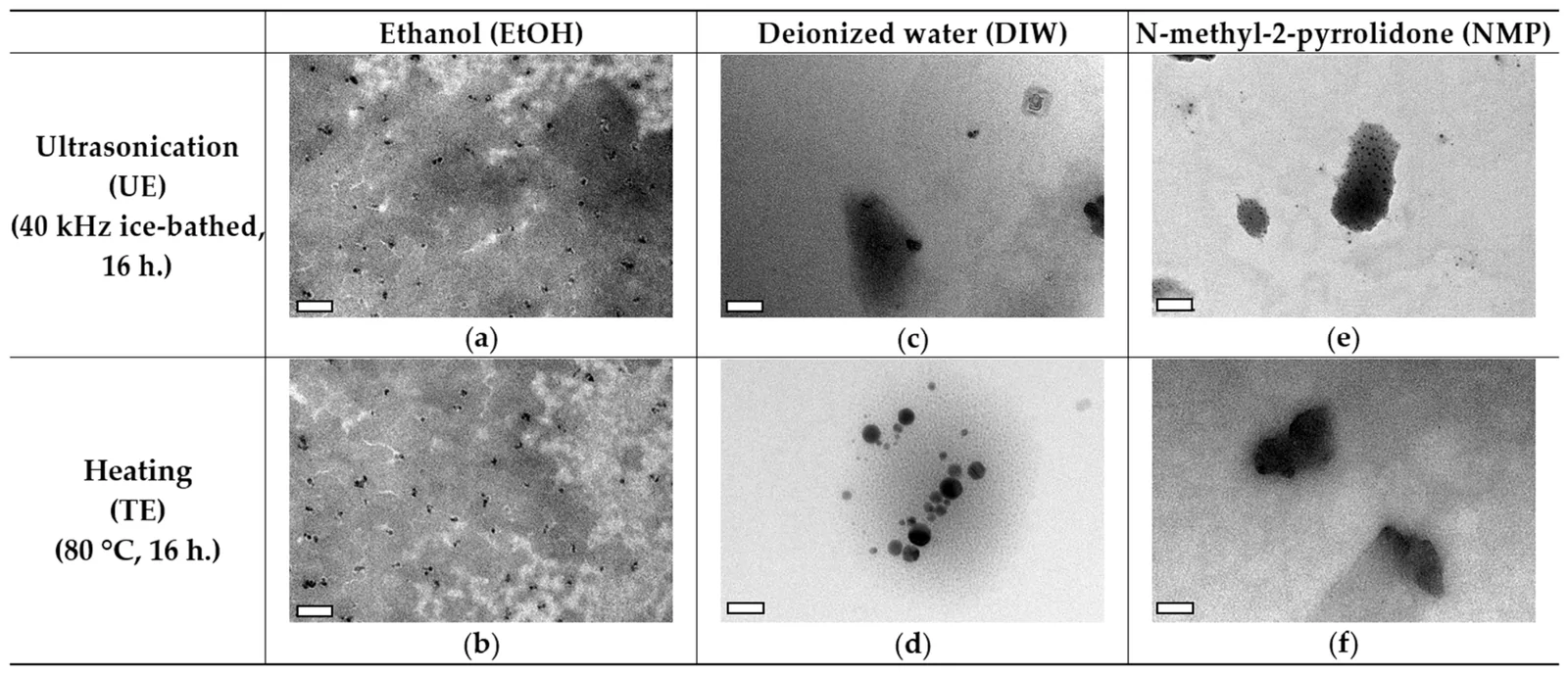
TEM image of MoS_2_ nanoparticles synthesized using different treatments and solvents; scale bar = 50 nm. (**a**) ×300K. (**b**) ×300K. (**c**) ×300K. (**d**) ×300K. (**e**) ×300K. (**f**) ×300K.

The morphology and diffraction pattern of MoS_2_ nanoparticles from TEM are shown in Figure 2, where MoS_2_ nanoparticles are visible as black dots. Only the ethanol (EtOH) (a) and (b) synthesized nanoparticles show some blurred rings, which may indicate the presence of small crystallites. The crystal structure is further examined by XRD in the next section.

**Figure 3:**
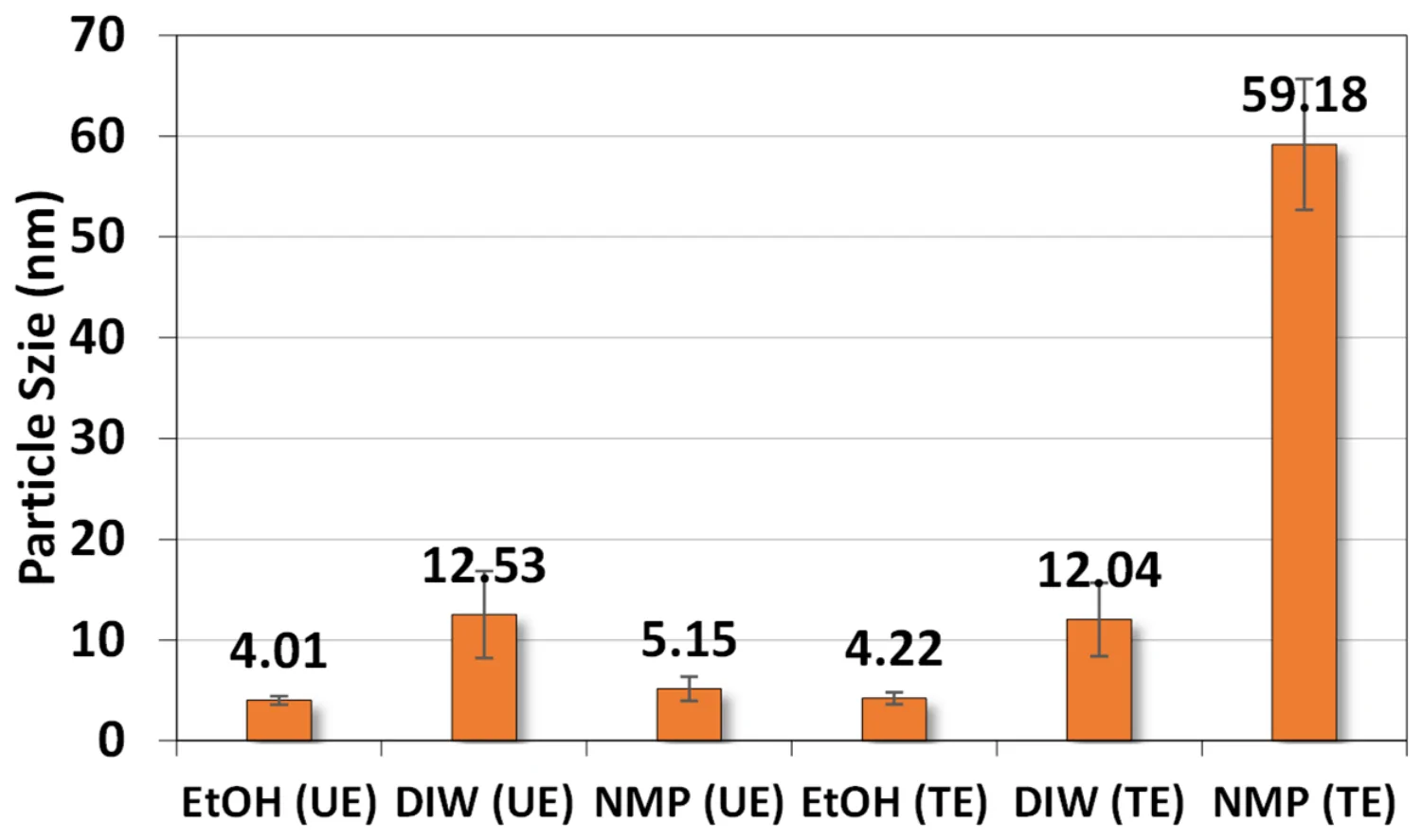
Size of MoS_2_ nanoparticles synthesized using different methods (UE: ultrasonic exfoliation; TE: thermal exfoliation) and solvents (EtOH: ethanol; DIW: deionized water; NMP: N-methyl-2-pyrrolidone) from TEM images by ImageJ^®^ (*n* ≥ 30).

The particle size of MoS_2_ nanoparticles was further analyzed in TEM images using ImageJ^®^ (*n* ≥ 30). As shown in Figure 3, ethanol is particularly effective in exfoliating the bulk MoS_2_ to around 4 nanometers, whether by sonication or thermal treatment. DI water yielded an average size of 12 nanometers, which may be attributed to the lower solubility of nanoparticles.

The significantly larger particle size of MoS_2_ achieved by thermal exfoliation in NMP is a surprise, as MoS_2_ in the same solvent under ultrasound treatment is relatively small (~5.15 nm). This could indicate that the MoS_2_ in NMP is less effectively exfoliated by heating. However, we retain this set of samples for further study because a larger size would exhibit different optical behaviors, as seen in later sections.

#### 3.2.2. XRD

**Figure 4:**
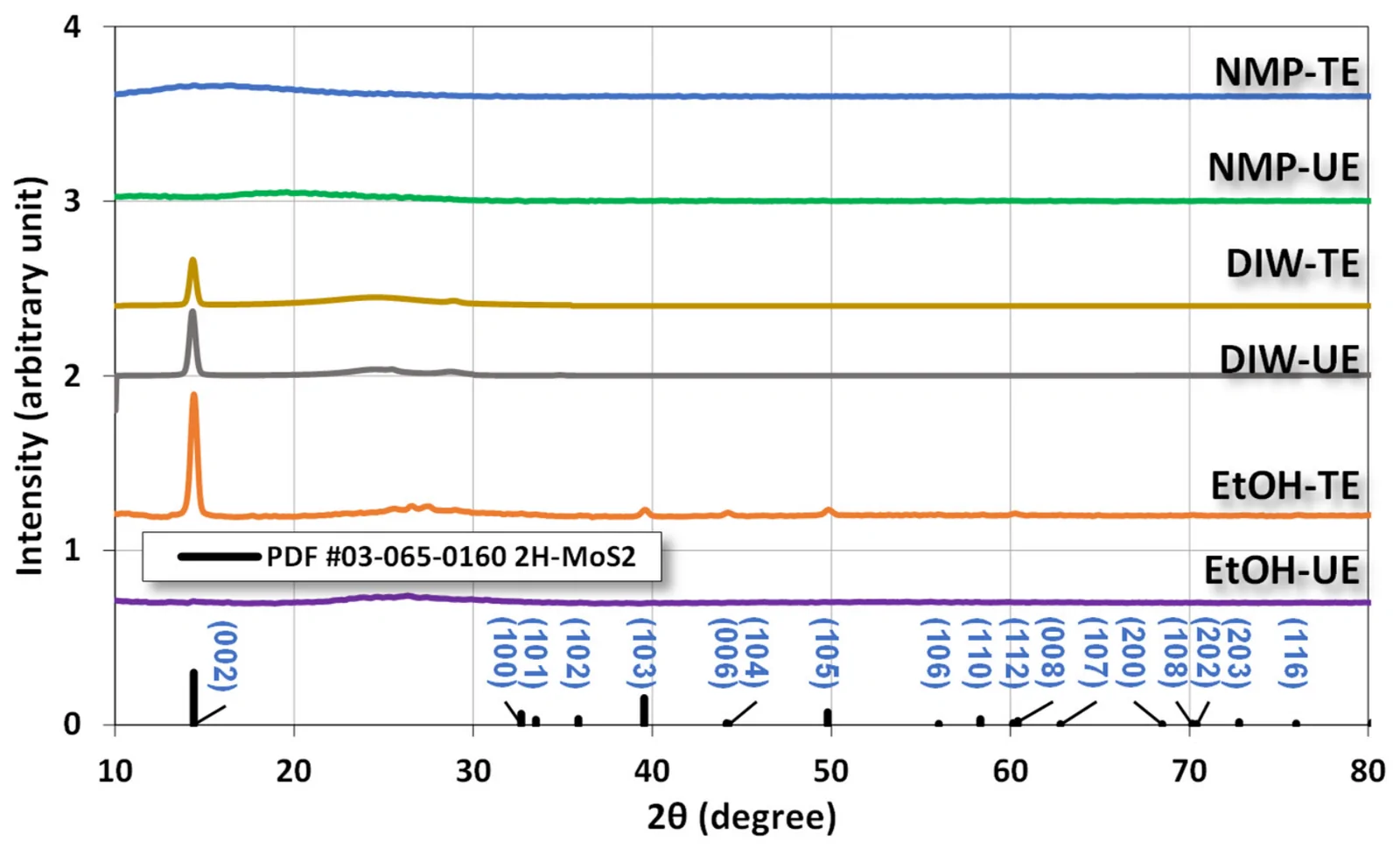
XRD of MoS_2_ nanoparticles synthesized using different methods (UE: ultrasonic exfoliation; TE: thermal exfoliation) and solvents (EtOH: ethanol; DIW: deionized water; NMP: N-methyl-2-pyrrolidone).

Figure 4 shows the crystal structures of MoS_2_ nanoparticles exfoliated using different methods and solvents. One dominant crystal plane (002), identified as 2H-MoS_2_ (PDF#03-065-0160), is clearly presented in the samples of ultrasonic exfoliation in DI water (DIW-UE) and thermal exfoliation in ethanol and DI water (EtOH-TE and DIW-TE). Thermal exfoliation in ethanol yields more crystal ODs than other methods. Other crystal planes of 2H-MoS_2_, such as (100), (103), (105), and (110), are relatively insignificant [61]. Samples synthesized in NMP are either amorphous-like or contain only crystallites.

The dominant (002) peak at 14.9° indicates a clean hexagonal structure (2H-MoS_2_) in the sample [62]. If more bulky structures such as nanosheets or large particles are present, other crystal planes would be more visible, possibly along with naturally occurring 1T-MoS_2_.

#### 3.2.3. Zeta Potential

The zeta potentials of MoS_2_ nanoparticles exfoliated by different methods and solvents are shown in Table 3. Samples exfoliated in DI water have lower absolute electrical potentials (less than 10 mV), implying unstable nanoparticle dispersion in the solvent. Over time, particle aggregation would lead to sedimentation in the solvent. On the contrary, with zeta potentials close to 30 mV or beyond, MoS_2_ nanoparticles can maintain suspension in ethanol or NMP for an extended time, even though the average particle size in NMP achieved by thermal exfoliation is much larger [63].

**Figure 5:**
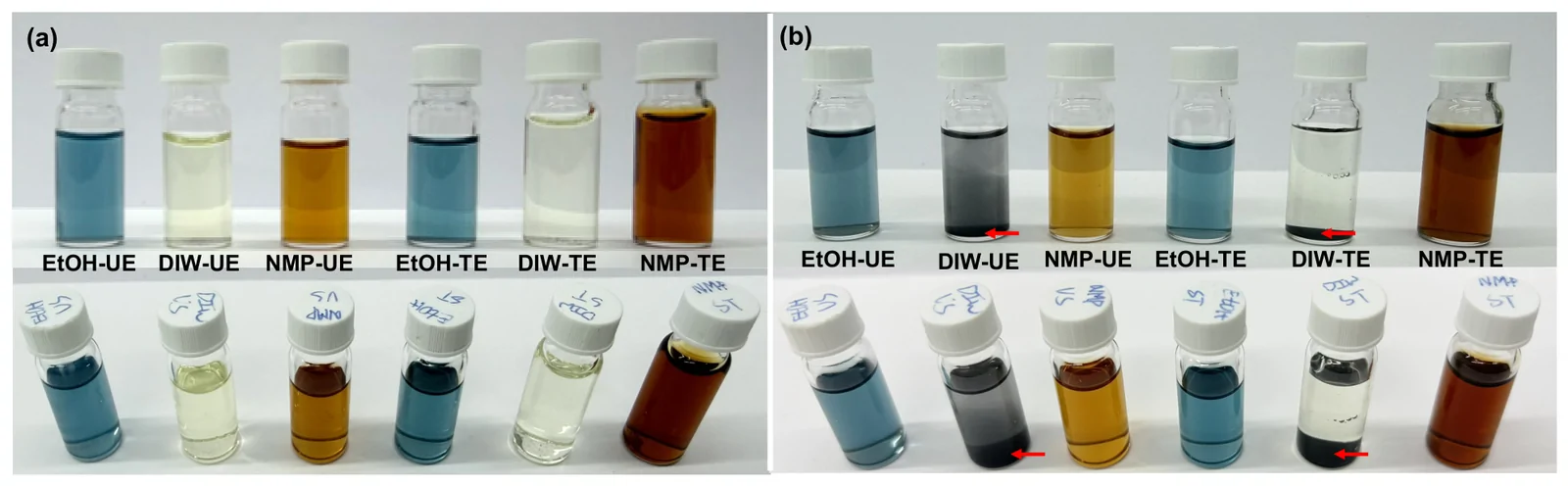
Photos of the MoS_2_ nanoparticles synthesized using different methods and solvents. (**a**) Day 1 and (**b**) day > 30; the sediments in DI water are marked with red arrows. (UE: ultrasonic exfoliation; TE: thermal exfoliation; EtOH: ethanol; DIW: deionized water; NMP: N-methyl-2-pyrrolidone).

Figure 5 shows a visualization of the colloidal state of exfoliated nanoparticles in different solvents, where stratification and sediments in DI water for samples stored longer than 30 days are visible and marked with red arrows. This observation is consistent with the zeta-potential measurement.

Another interesting but self-explanatory observation in Figure 5a is that the color of these suspensions is consistent with their respective UV–visible–NIR spectra.

#### 3.2.4. XPS

**Figure 6:**
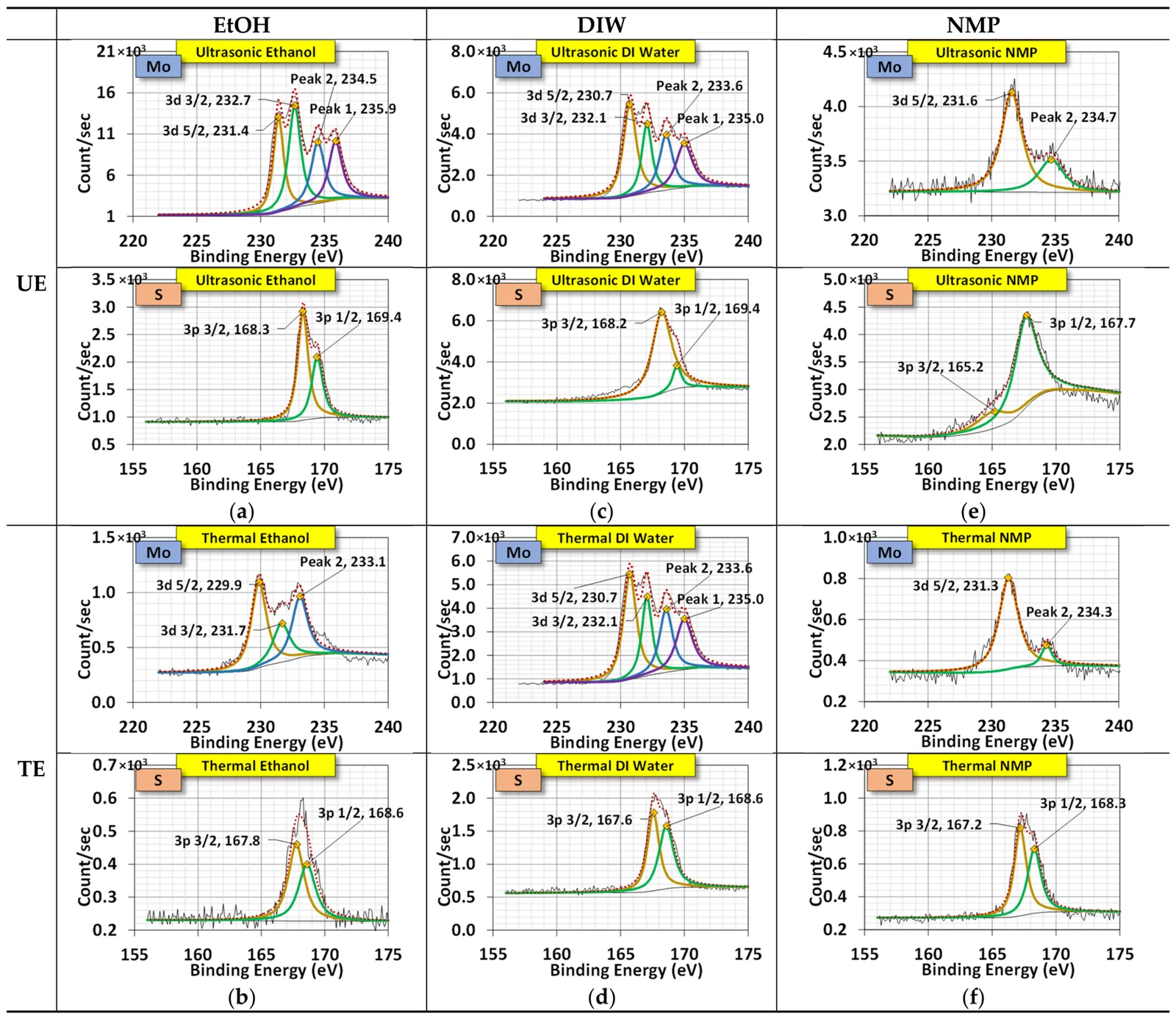
XPS of MoS_2_ nanoparticles synthesized using different methods and solvents. (**a**) Ultrasound in EtOH; (**b**) Thermal in EtOH; (**c**) Ultrasound in DI water; (**d**) Thermal in DI water; (**e**) Ultrasound in NMP; (**f**) Thermal in NMP.

Figure 6 presents the XPS for the binding energy spectra of MoS_2_ nanoparticles synthesized under different exfoliations in different solvents. The Gaussian function provided a good fit to the spectra for both Mo and S.

Starting from sulfur, both 3p_3/2_ and 3p_1/2_ peaks are numerically found at around 167.6–168.1 eV, regardless of the exfoliation method or solvent type. These two bonding energy levels of S indicates that S can form a stable bond with Mo.

The bonding of Mo, on the contrary, is more complicated. Core-level peaks at 3d_5/2_ (approximately 229–231 eV) and 3d_3/2_ (approximately 232 eV) correspond to the normal bonding states of Mo. The other two peaks at higher energy levels—namely, peak 2 (approximately 233–234 eV) and peak 1 (approximately 235 eV)—are satellite peaks, most commonly caused by interactions between excited core and valence electrons. The interactions can either promote valence electrons to higher energy levels (shake-up) or eject them from the atom (shake-off). Both result in lower kinetic energy of the emitted photons and a binding-energy shift higher than that of core-level peaks.

However, the low crystallinity indicated by the XRD (Figure 4) also indicates the bonding state of Mo and S. Because the crystal structures are not perfectly ordered due to exfoliation, defects (vacancies, dislocations, grain boundaries, etc.) are inevitable within these incomplete structures. More details about the imperfections of the MoS_2_ structure shall be discussed later.

#### 3.2.5. UV–Visible–NIR Spectrum

**Figure 7:**
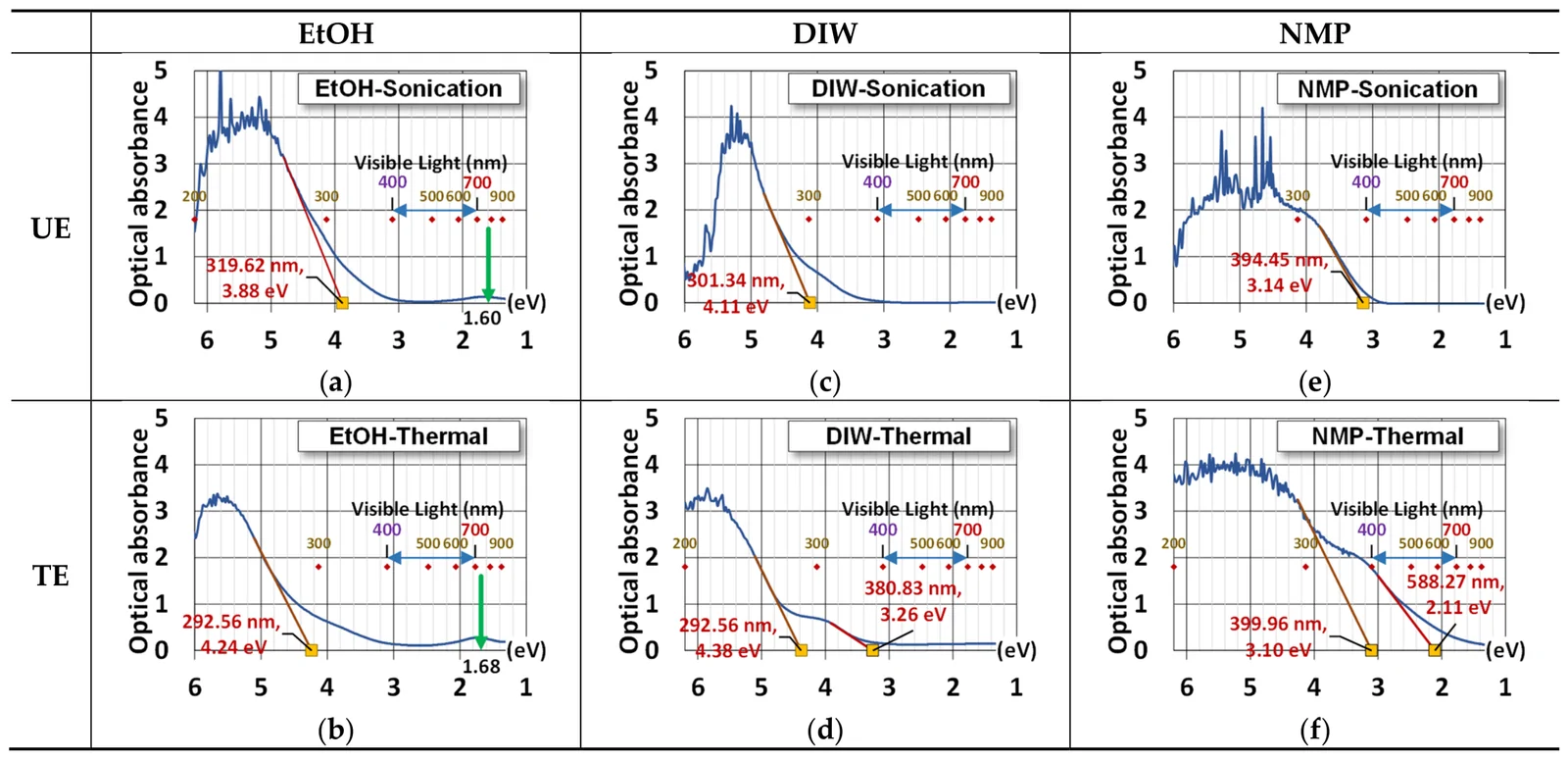
UV–visible–NIR spectra of MoS_2_ nanoparticles synthesized using different methods and solvents. (**a**) Ultrasound in EtOH; (**b**) Thermal in EtOH; (**c**) Ultrasound in DI water; (**d**) Thermal in DI water; (**e**) Ultrasound in NMP; (**f**) Thermal in NMP. The estimated minimum energy of optical absorption is defined as the intersection of a linear extrapolation from the absorption edge with the abscissa. A broad absorption around 700 nm is also labeled by green arrows for MoS_2_ nanoparticles synthesized in ethanol in (**a**,**b**).

Figure 7 shows the optical absorption of UV–visible–NIR spectra. For all samples in DI water, broad UV absorption is observed, regardless of the synthesis method. However, this broadband peak can be roughly categorized into two sub-band peaks for samples synthesized by ultrasonication. One is around 4.5 eV (270 nm) due to photon-induced excitons in MoS_2_ nanoparticles, and the other is between 5.0 and 5.5 eV (247.96–225.42 nm) due to photons interacting with quasi-continuous electronic band structures arising from geometric confinement [64]. Thermally exfoliated samples show broadband absorbance at slightly higher energy levels around 5.5–6.0 eV (225.42–206.64 nm).

Notably, samples synthesized in ethanol show another broadband absorption centered around 1.68–1.60 eV (737–776 nm) (Figure 7a,b). This peak is related to the photothermal effect, in which energy from absorbed photons is released at longer (red-shifted) wavelengths. Thus, heat can be generated through the interactions between electrons and phonons (lattice vibrations) [65,66].

Given the UV–visible–NIR spectra, we can estimate the minimum absorption energy of the synthesized MoS_2_ nanoparticles by linear extrapolation from the absorption edge. As shown in Figure 7, the *first intersections* with the horizontal axis (minima of absorption energy) are all in the UV range (shorter than 400 nm, or 3.0996 eV). In other words, electrons in these particles are excitable if photons provide UV-level energy to help them jump over the band gap. Although this estimate is very rough, the widened band gaps in MoS_2_ nanoparticles are illustrative compared with their bulk forms (approximately 1.2 eV) [67,68,69,70,71,72].

Uniquely, samples synthesized by thermal exfoliation in NMP and DI water ((d) and (f)) present a *second intersection* with the horizontal axis. This intersection indicates another absorption in visible light. The spectrum of nanoparticles synthesized in NMP is a gradually decreasing absorption stretching from the UV region to the NIR region. This distinct pattern is more similar to that of a continuous phase, such as thin films [73,74], and can be attributed to the larger MoS_2_ particle size, as shown in the TEM image in Figure 3.

#### 3.2.6. Photoluminescence

**Figure 8:**
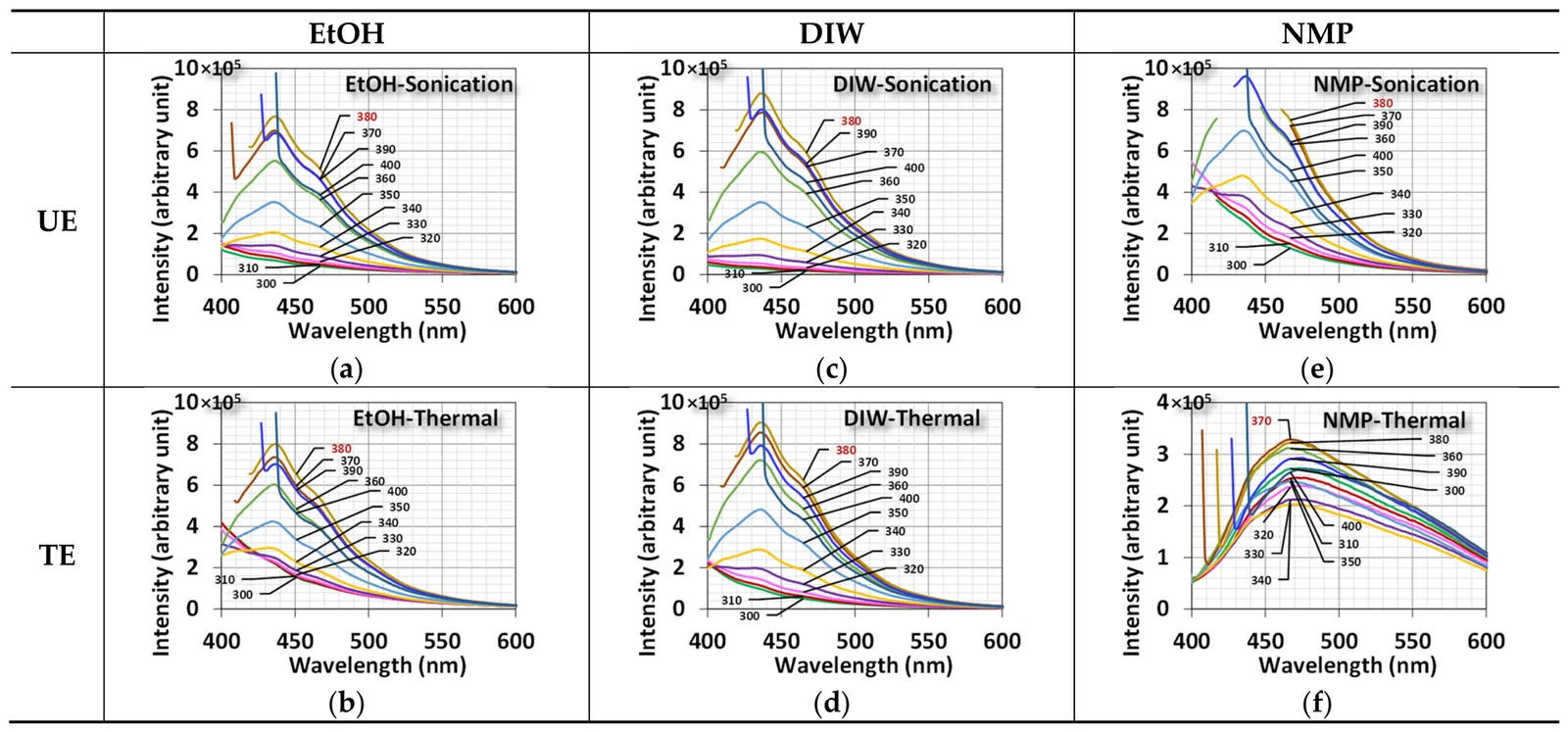
Photoluminescence spectra of MoS_2_ nanoparticles synthesized using different methods and solvents. The wavelength of laser excitation is marked by numbers, and the one that produces the highest overall intensity for each case is marked in red. (**a**) Ultrasound in EtOH; (**b**) Thermal in EtOH; (**c**) Ultrasound in DI water; (**d**) Thermal in DI water; (**e**) Ultrasound in NMP; (**f**) Thermal in NMP.

Figure 8 shows the photoluminescence of synthesized MoS_2_ nanoparticles in DI water under different laser excitation wavelengths. Except for the synthesis in NMP by heating, the peak of optical emission from samples was found to be centered around 440–450 nm (2.82–2.76 eV), regardless of the type of solvent and treatments, and laser excitation of 370–380 nm (3.35–3.26 eV) produced the highest emission among all cases.

More specifically, when the laser excitation wavelength is longer than 340 nm (3.65 eV), luminescent emission intensifies. Except for samples synthesized in NMP by heating, the emission reaches a maximum at varying wavelengths between 440 and 450 nm, then decreases, highlighting a resonance effect. All the decreasing emissions show a shoulder around 470 nm (2.64 eV), indicating possible intra-band transitions during electron relaxation [75,76,77,78].

In the case of NMP thermal exfoliation, broad photoluminescence covers almost the whole visible spectrum. This result is due to a larger particle size (Figure 3). In other words, the photoluminescence diminishes as the quantum confinement weakens when particles become larger [79].

### 3.3. Photothermal Test

**Figure 9:**
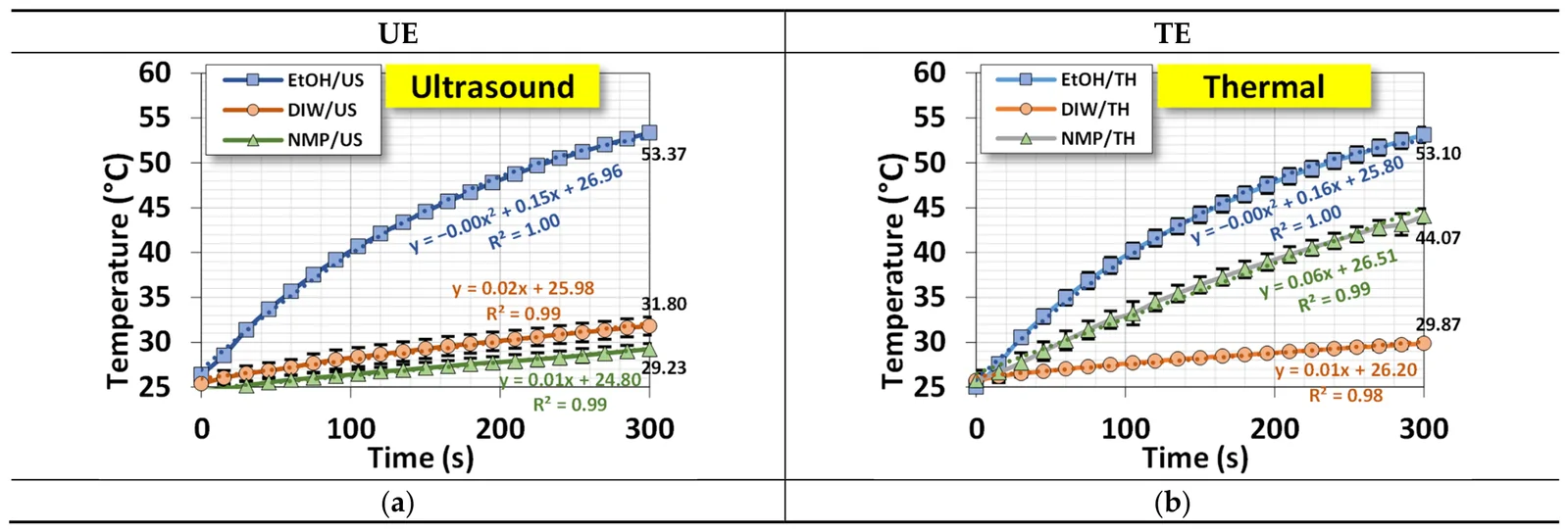
Temperature variation of MoS_2_ nanoparticles dispersed in DI water for different syntheses. The excitation laser wavelength is 808 nm. Exfoliation methods are denoted as either thermal or ultrasound. (**a**) Ultrasonic exfoliation; (**b**) Themal exfoliation.

The UV–visible–NIR absorption spectra showed a broad band centered at 737–776 nm for samples treated with ethanol. This absorption is helpful for clinical treatment if its function is utilized correctly [80]. Figure 9 shows the temperature change in DI water containing dispersed MoS_2_ nanoparticles as it is continuously exposed to an 808 nm LED laser. The most prominent temperature rise is observed with MoS_2_ nanoparticles synthesized in ethanol, which reach 53.1 °C (ultrasonic) and 53.37 °C (thermal) in 300 s. For the same period, other samples have a smaller temperature rise of less than 30 °C.

The photothermal effect usually occurs when excited electrons relax and interact with the lattice in the red-to-infrared region. In this range of wavelengths, the UV–visible–NIR spectra (Figure 7) of samples synthesized in ethanol indicate that photon energy is readily absorbed. The smaller MoS_2_ nanoparticles also improve energy conversion efficiency (Figure 3).

The thermal exfoliation of MoS_2_ nanoparticles in NMP was evident, with a higher photothermal effect (Figure 9b, temperature rising to 45 °C in 300 s). This result corroborates the broad optical absorption across the visible–infrared region (Figure 7f), thereby enhancing photothermal conversion [81].

### 3.4. In Vitro Cell Viability

**Figure 10:**
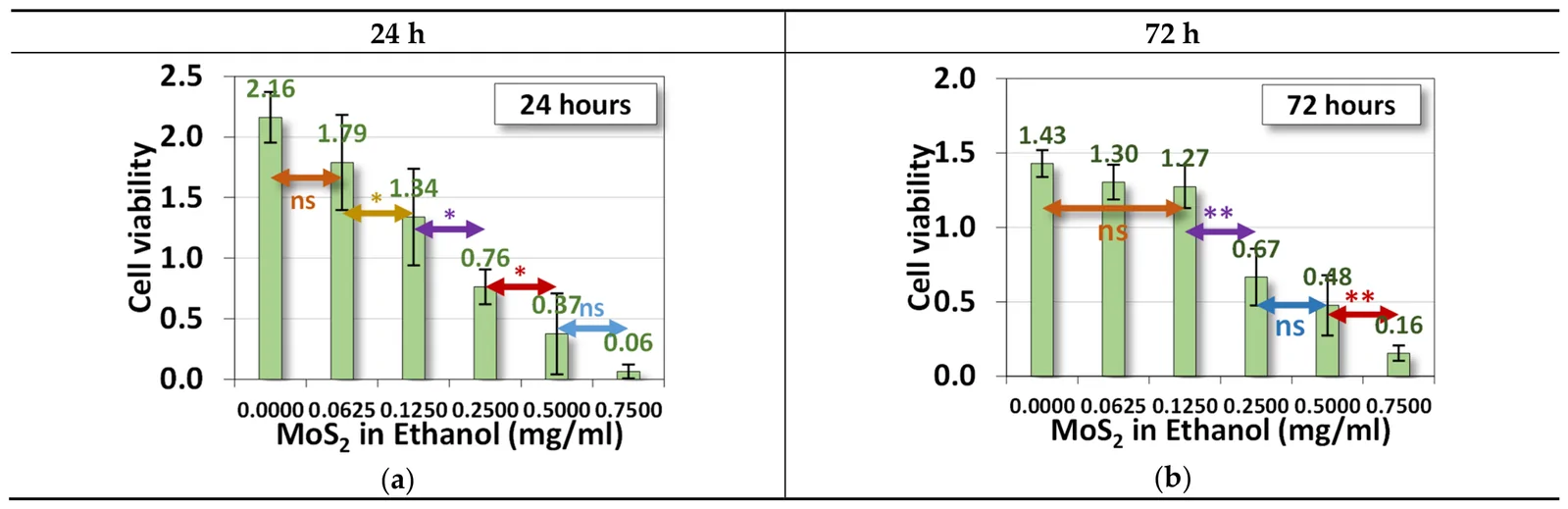
Viability of NIH/3T3 fibroblasts for in vitro cell culture with MoS_2_ nanoparticles synthesized in ethanol by heating after (**a**) 24 h and (**b**) 72 h of co-culture incubation. (*p*-value: **: 0.01, *: 0.05, ns: not significant; error bars represent the sample standard deviation, *n* = 6).

Figure 10 shows the relative cell viability of NIH/3T3 fibroblast cells in vitro at 24 and 72 h after seeding. Only MoS_2_ nanoparticles synthesized in ethanol by thermal exfoliation were chosen for the in vitro cell culture because of their highest photothermal effect among the samples. In the medium mixed with MoS_2_ nanoparticles, viability decreases as MoS_2_ concentration increases. However, the low concentrations of 0.0625 and 0.125 mg/mL showed minimal toxicity (viability > 100%) in NIH/3T3 fibroblast cultures, particularly after 72 h, when cells were stabilized and began to proliferate again. The relative cell viability of these two concentrations remained above 100% 72 h after seeding.

### 3.5. Antibacterial Test

**Figure 11:**
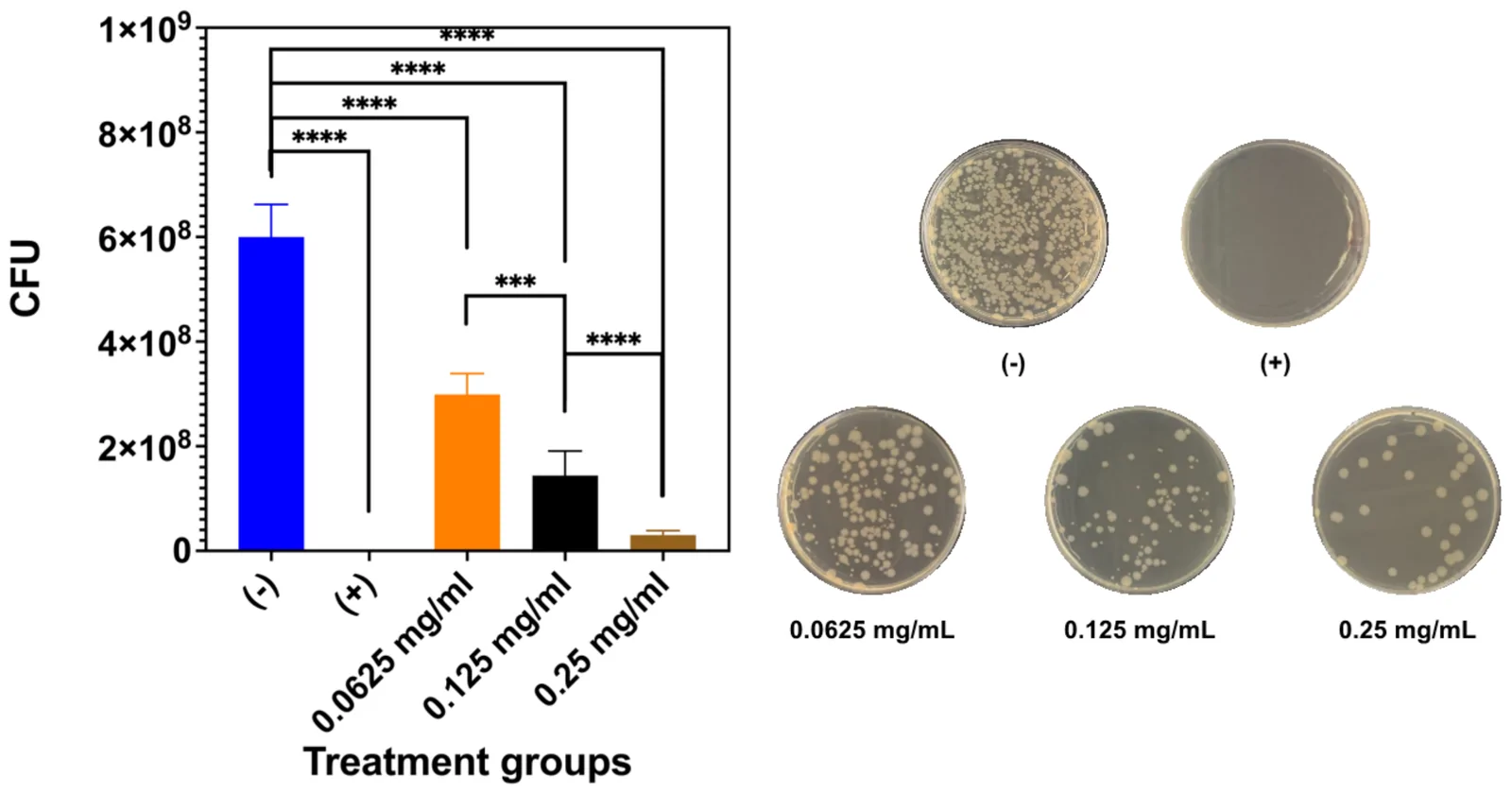
CFU counting (24 h) against *E. coli* by MoS_2_ nanoparticles synthesized using different methods (UE: ultrasonic exfoliation; TE: thermal exfoliation) and solvents (EtOH: ethanol; DIW: deionized water; NMP: N-methyl-2-pyrrolidone). The control group (−) is the culture media with *E. coli* only, and the positive control (+) is the medium with penicillin–streptomycin. (****: *p* < 0.0001, ***: *p* < 0.001; error bars represent the sample standard deviation, *n* = 3).

Figure 11 shows CFU counts of *E. coli* 24 h after co-culture with MoS_2_ nanoparticles in culture medium. Only the 0.25, 0.125, and 0.0625 mg/mL concentrations of nanoparticles were selected for this test, based on the in vitro biocompatibility of MoS_2_ nanoparticles. The antibacterial effect increases with MoS_2_ concentration. Due to the optical absorption of MoS_2_ nanoparticles, they exhibit higher absorbance than the control group at early time points (Figure 11. The 0.25 mg/mL concentration of MoS_2_ nanoparticles effectively suppressed *E. coli* proliferation, reducing CFU counts by nearly 1/10.

Note that both the in vitro biocompatibility and antibacterial tests are preliminary but quantitative. The underlying mechanisms of imposed toxicity appear to point to endocytosis, but this is beyond the scope of the current study.

## 4. Discussion

### 4.1. The Estimated Bandgap Energy

**Figure 12:**
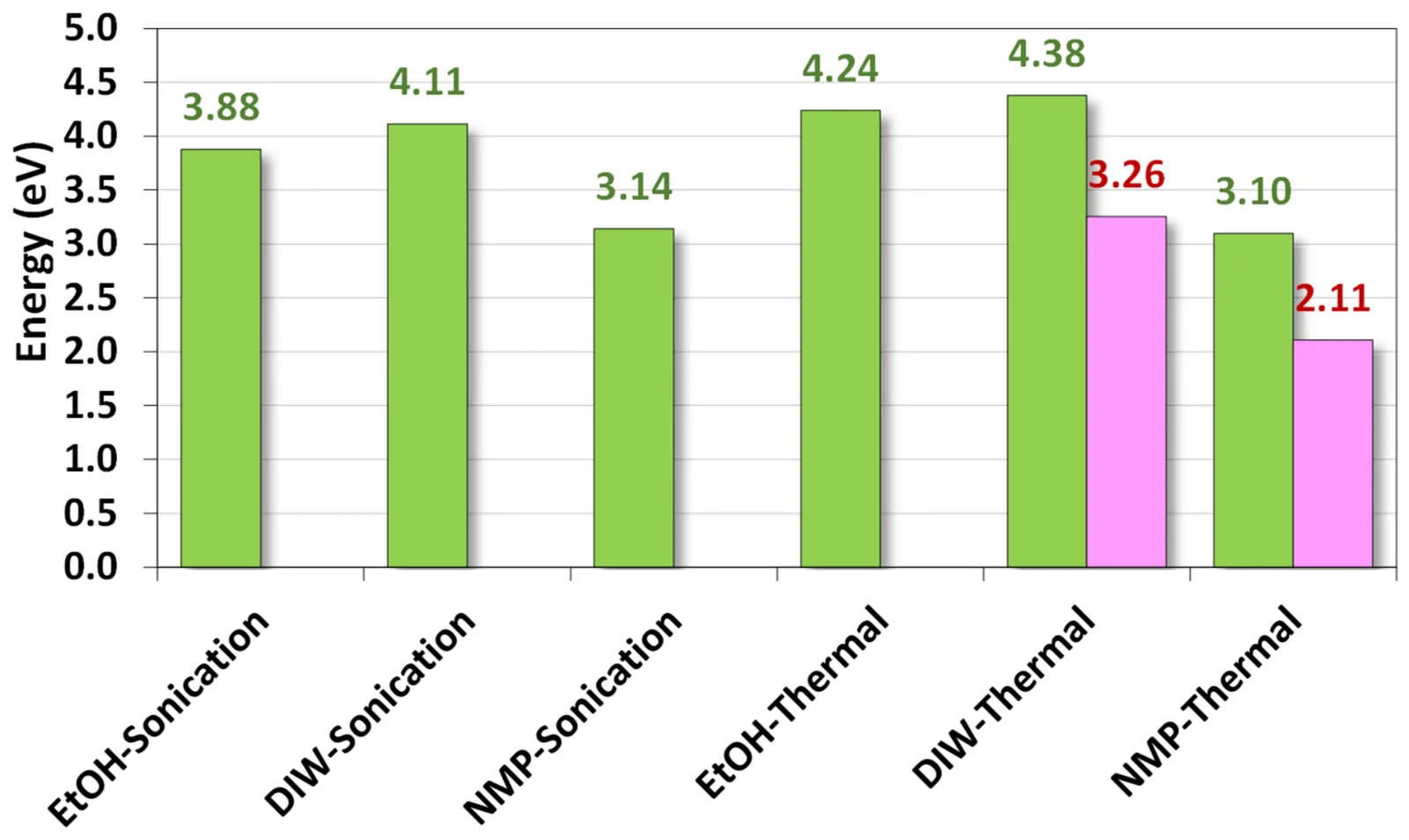
Summary of estimated band gaps in MoS_2_ nanoparticles synthesized using different exfoliation methods and solvents.

The differences in band gaps are due to the geometric confinement effect, which widens the band gap as size decreases. This inverse relationship is clearly shown in this study. Figure 12 compares estimated band gaps from UV–visible–NIR spectra of MoS_2_ nanoparticles synthesized with different solvents and via exfoliation. These higher-energy band gaps require photons with shorter wavelengths to excite electrons to the conduction band. Note that thermal exfoliation in DI water and NMP produces a second, smaller band gap in nanoparticles (3.26 eV at 380.32 nm and 2.11 eV at 587.60 nm, respectively), as shown by the linear intersection lines in Figure 7. These gaps may imply the existence of intra-bands in the microstructure.

A freestanding monolayer MoS_2_ is predicted to have direct band gaps around 1.8–2.8 eV, and the bulk MoS_2_ (hexagonal and rhombohedral symmetry) showed narrower indirect band gaps around 1.2 eV [67,68,69,70,71,72]. Given the multiple band gaps associated with polycrystallinity in nanoparticles, the estimated band gap from our UV–visible–NIR spectra falls within the upper bound of findings from other studies.

### 4.2. Correspondence Between XPS and XRD for the Microstructure

From XRD in Figure 4, we found the only dominant crystal plane in the samples is (002) of 2H MoS_2_. The 2H MoS_2_ has the following bonding features [82,83,84]:

- Mo 3d peaks: Pristine semiconducting 2H-MoS_2_ exhibits a Mo 3d_5/2_ peak at ~229.3 eV and Mo 3d_3/2_ at ~232.5 eV, denoting a stable Mo^4+^ oxidation state. The binding energies of Mo in XPS (Figure 6a,b,d,e) for samples exfoliated in ethanol and DI water almost match these two energy levels, confirming the presence of Mo^4+^.
- S 2p peaks: The corresponding S 2p_3/2_ and S 2p_1/2_ spin-orbit components for pristine 2H-MoS_2_ appear at ~161.6 to −162.2 eV and ~162.8 to 163.4 eV, respectively, representing covalent metal–sulfur hybridization within the trigonal prismatic coordinate layers. The S bonding energies in XPS (Figure 6) for samples exfoliated in three solvents all shift higher, indicating defects in S, besides its regular bonding to Mo.
- Impurities or defects in S also affect the Mo bonding energy, and the satellite states reflect this fact.

Based on this information from XRD and XPS, we can characterize the main cause of disorder or amorphousness as missing or displaced S atoms during exfoliation, which is physically reasonable, as S is much smaller than the central metal, Mo and, thus, disulfide bonds can be disrupted or broken much more easily.

NMP effectively destroyed the orderliness of MoS_2_ microstructures compared to ethanol or DI water. This likely explains why only amorphousness was detected in its XRD.

### 4.3. Estimate of Quantum Yield from Photoluminescence

As shown by the photoluminescence spectroscopy in Figure 7, the majority of emission peaks are at 440–450 nm, corresponding to an energy of *E* = *hc*/*λ* = 2.8178–2.7552 eV. This is in the realm of violet-blue light. However, most samples exhibit the highest luminescence intensities under 380 nm laser irradiation (3.2627 eV). Thus, the *energy loss* or absorption between the excitation and emission peaks is around Δ*E* ≈ 0.4449–0.5075 eV. For imaging purposes, this is quite a decent energy conversion ratio [85,86,87].

**Figure 13:**
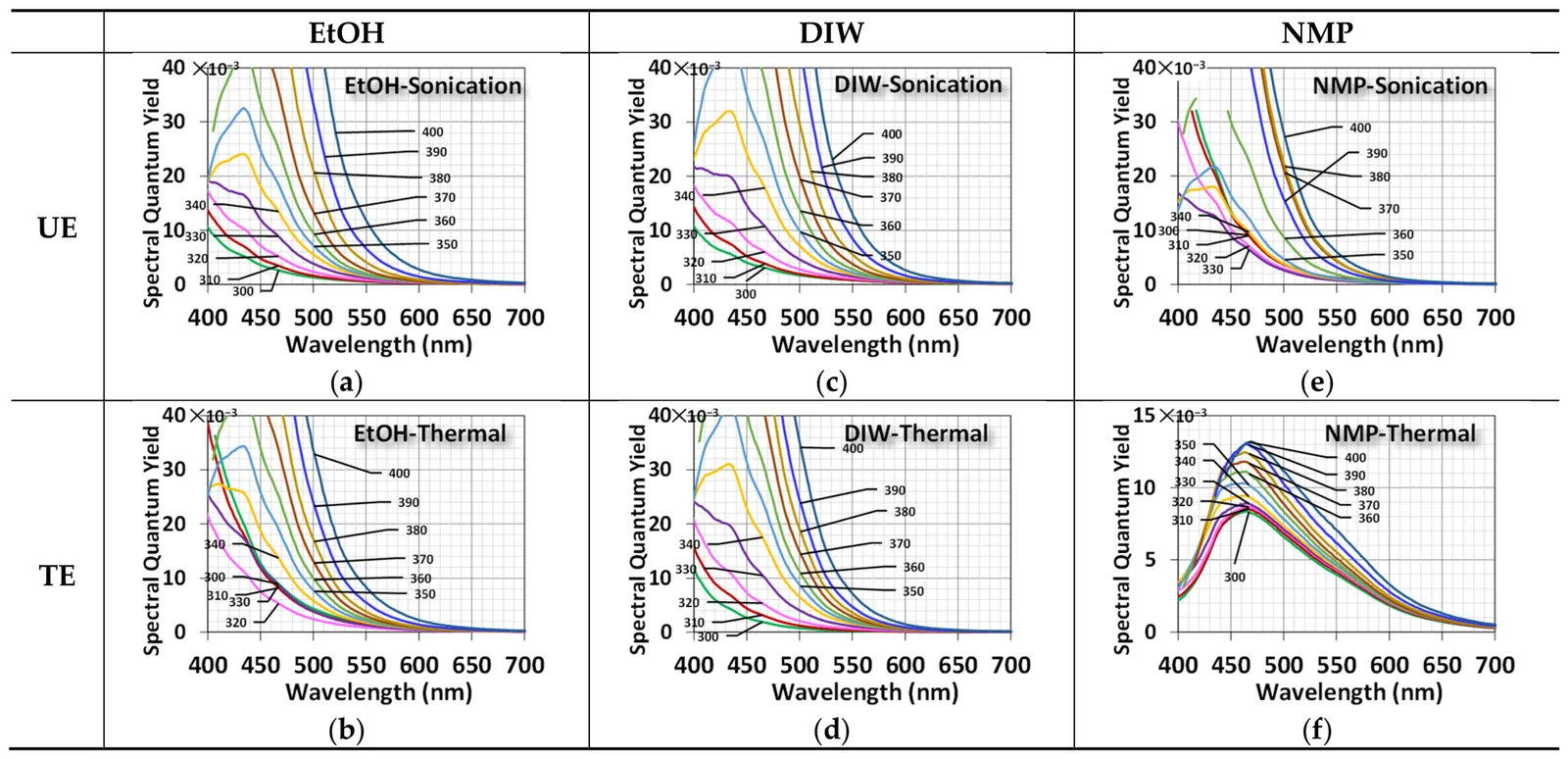
The spectral quantum yield from photoluminescence for MoS_2_ nanoparticles synthesized in various solvents and by different exfoliation methods (**a**) Ultrasound in EtOH; (**b**) Thermal in EtOH; (**c**) Ultrasound in DI water; (**d**) Thermal in DI water; (**e**) Ultrasound in NMP; (**f**) Thermal in NMP.

In fact, the quantum yield (QY) or energy ratio between the irradiation and photoluminescence of a certain wavelength (*λ* or *λ_i_*) can be calculated as follows [88,89,90]:

(7) $${QY}_{\lambda }=\frac{energy\;of\;emission}{energy\;of\;absorption}=\left(\frac{Intensity\left(\lambda \right)E_{\lambda }d\lambda }{\int Intensity\left(\lambda \right)E_{\lambda }d\lambda }\right)\left(\frac{E_{\lambda }}{E_{\lambda_{irradiation}\;*\;optical\;absoranvce\;}}\right)$$

where *E_λ_* (=*hc*/*λ*) is the photon energy of wavelength *λ*; *h* and *c* are the Planck constant and speed of light, respectively; and *λ*_irradiation_ is the wavelength of the incident laser. The term $\left(\frac{Intensity\left(\lambda \right)E_{\lambda }d\lambda }{\int Intensity\left(\lambda \right)E_{\lambda }d\lambda }\right)$ is the probability of *E_λ_*. The integral can be calculated numerically using Riemann sums with the trapezoid rule, and the resulting spectral quantum yield is shown in Figure 13. In all samples except those exfoliated in NMP, the pattern of yield under laser wavelengths between 300 and 330 nm shows decay only in the visible spectrum. When irradiated with lasers at 340–400 nm, a peak in quantum yield is observed at 440–450 nm.

The thermally synthesized nanoparticles in NMP all have peaks between 450 and 500 nm and decay slowly across the visible spectrum. The quantum yields also increase with the laser irradiation wavelength, i.e., the longer the wavelength, the higher the yield.

We can further calculate the average quantum yield from a specific laser irradiation (QY*_λ_*_iradiation_) using Equation (8) as follows.

(8) $$E\left({QY}_{\lambda_{irradiation}}\right)=\frac{\int {QY}_{\lambda }d\lambda }{\int d\lambda }$$

**Figure 14:**
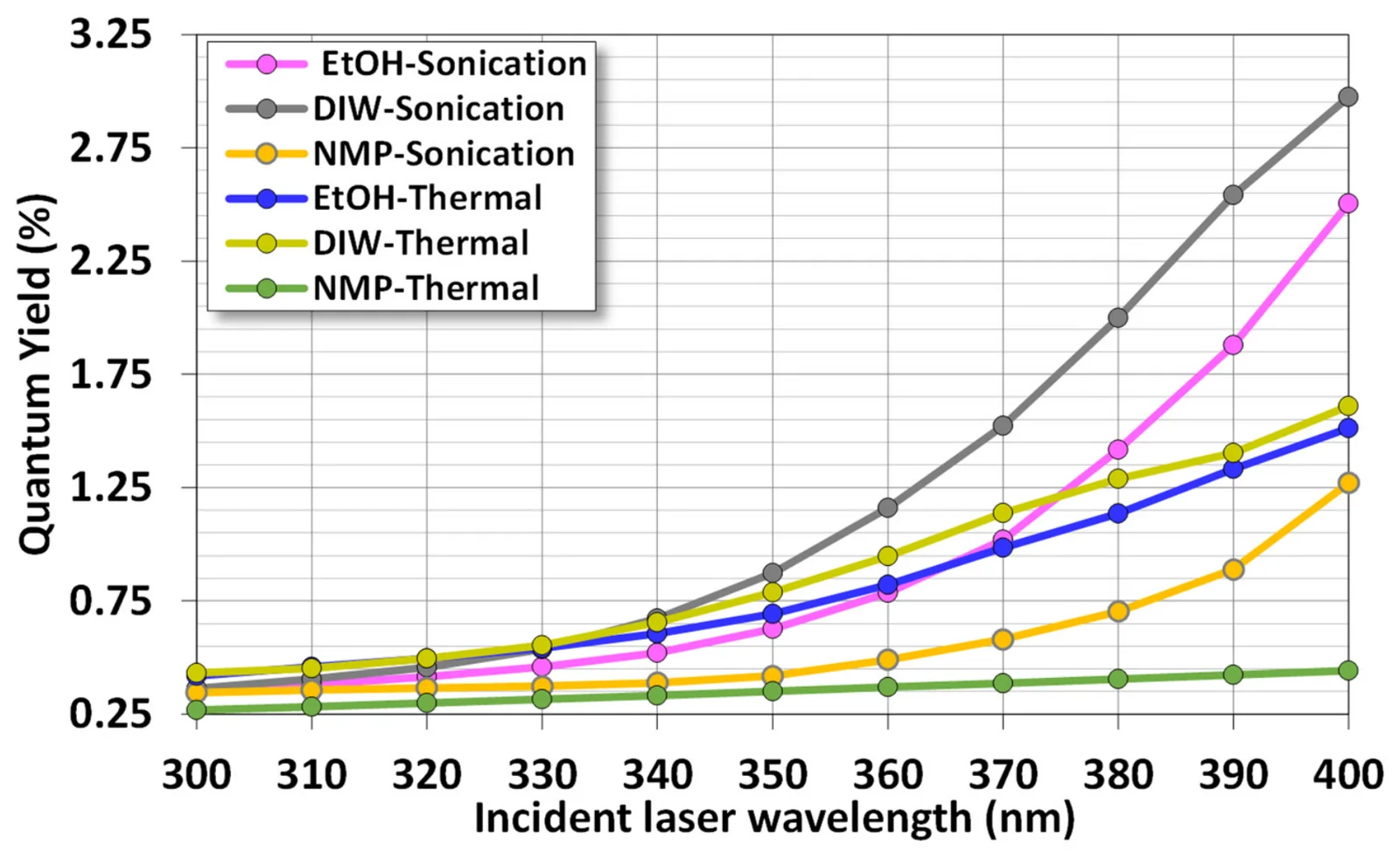
The quantum yield for specific irradiation laser wavelengths from photoluminescence for MoS_2_ nanoparticles synthesized in various solvents and by different exfoliation methods.

Figure 14 shows results of the calculation; the average yield increases with longer excitation laser wavelength and ranges from 0.25% to 2.25%. This range is typical of photoluminescence quantum yields of non-specially treated MoS_2_ [36]. Thermally exfoliated nanoparticles in NMP increase only slightly to 0.44% under 400 nm laser irradiation, reflecting their much larger particle size and limited dimensional confinement for quantum effects.

### 4.4. Evaluation of Heat Generation in Photothermal Test from Finite Element Analysis

The time average of a piecewise continuous function of the heat source used in the finite element analysis (Figures S2 and S3) to obtain an optimal solution to the photothermal measurement can be calculated as

(9) $${Heat\;soruce}_{average}=\frac{\int_{0}^{300}Heat\;soruce\left(t\right)dt}{300}.$$

**Figure 15:**
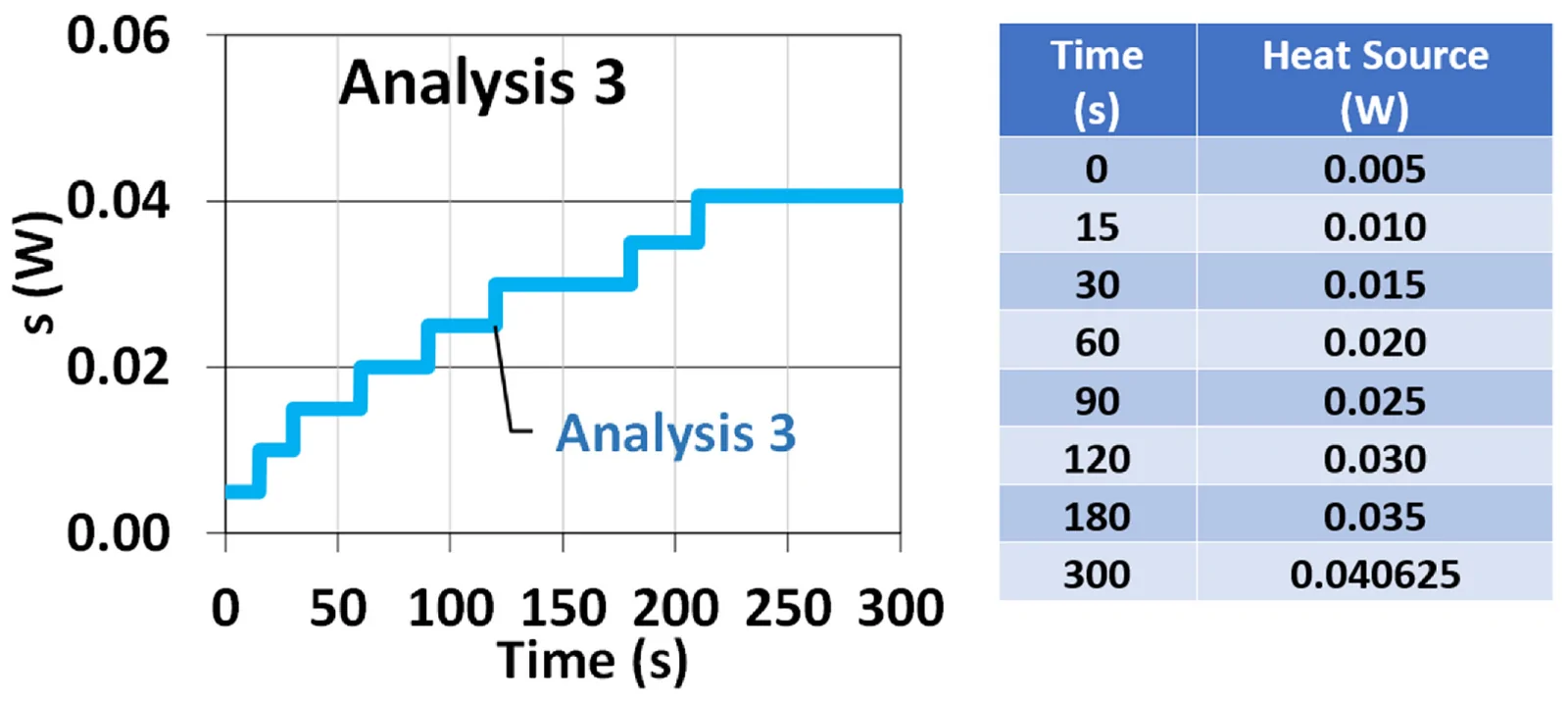
A piecewise-continuous representation of the heat source is required for optimal results when comparing finite element analysis with photothermal experimental measurements.

Using the piecewise-continuous function shown in Figure 15, the average of the heat source is about 0.028432 W. Since we only use an octant volume of the domain for calculation (Figure S1), the total power absorbed by the whole volume under an 808 nm laser (1 W) irradiation would be eight times larger, that is, 0.22746 W. In other words, the utilization of the laser power absorbed by nanoparticles to heat the water would be around 22.75% (0.22746 W/1 W). In view of such a simplified approximation, this is quite a reasonable estimation when compared to the absorption measured by a UV–visible–NIR spectrometer, in which the ratio of absorption is 1 − (*I_T_*/*I*_0_) ≈ 1–10^(−*A*)^ = 1–10^(−0.13)^ = 25.86%.

### 4.5. Evaluation of Fabricated MoS_2_ Nanoparticles

We selected exfoliated MoS_2_ nanoparticles in ethanol as an example for comparison with results from other studies. This selection is based on their higher photothermal heating in DI water (Figure 9b). The comparison includes production yield, particle size, photoluminescence quantum yield, and photothermal effects, as listed in Table 4. Overall, the syntheses of MoS_2_ nanoparticles conducted in this study are comparable in terms of particle size, exhibit higher photothermal efficiency (as measured in DI water), and consume less energy during exfoliation.

## 5. Conclusions

An economic, one-pot synthesis of MoS_2_ nanoparticles at atmospheric pressure was investigated using ultrasonic or thermal exfoliation at 80 °C in three solvents: ethanol (EtOH), N-Methyl-2-pyrrolidone (NMP), and DI water (DIW). The highest production yield of MoS_2_ nanoparticles is achieved in EtOH by either ultrasound or thermal exfoliation due to the higher solubility of MoS_2_ (approximately 0.1–5 mg/mL), which leads to a higher exfoliation rate. The UV–visible–NIR spectra of synthesized MoS_2_ nanoparticles in DI water showed a broad absorption peak around 340 nm due to photon-induced excitons. A smaller, broader absorption peak around 737–776 nm was also observed for samples synthesized in EtOH. This absorption is associated with photothermal excitation. The photoluminescence of MoS_2_ nanoparticles in DI water exhibited the highest emission intensity, at 440–450 nm, with a shoulder peak around 470 nm upon excitation at 380 nm. This red shift in fluorescence emission indicates a two-stage relaxation of excited electrons after optical absorption. The temperature variation of DI water containing nanoparticles under 808 nm LED laser irradiation can rise to approximately 50 °C in 300 s if MoS_2_ nanoparticles were synthesized in EtOH. This result is consistent with the broad optical absorption in UV–visible–NIR spectroscopy. The viability of in vitro NIH/3T3 fibroblast cultures indicated that low concentrations (0.0625 and 0.125 mg/mL) of MoS_2_ nanoparticles synthesized in EtOH by thermal exfoliation are non-toxic or lethargic, allowing for NIH/3T3 fibroblast cell proliferation over 72 h.

In summary, this study reports a scalable, one-pot synthesis of MoS_2_ quantum dots under atmospheric conditions, without any intermediate interruptions during exfoliation. Solvent–thermal exfoliation was carried out at 80 °C, resulting in relatively low energy consumption. This temperature range contrasts with many other studies, which are usually conducted at 150 °C to 270 °C in high-pressure autoclaves, making them more scalable and safer for large-scale production. The MoS_2_ nanoparticles synthesized in ethanol exhibit a unique NIR absorption band, indicating their dual function of photothermal conversion and photoluminescence. Finally, we propose a simple yet effective numerical scheme to determine the heat-source term for finite element simulation of the photothermal test.

## Figures and Tables

**Table 1:** Solvent combinations and exfoliation methods for the experimental design *.

	Ethanol (EtOH)	Deionized Water (DIW)	N-Methyl-2-Pyrrolidone (NMP)
**Ultrasonication (UE)** **(40 kHz ice-bathed, 16 h.)**	20 mL	20 mL	20 mL
**Heating (TE)** **(80 °C, 16 h.)**	20 mL	20 mL	20 mL

* 200 mg MoS_2_ nanoparticles (dry mass) were added to all solvents, measured by an analytical balance.

**Table 2:** The yield of different synthesis methods and solvents for molybdenum disulfide nanoparticles.

Synthesis	MoS_2_ + Solvent	Product Weight (MoS_2_ Nanoparticles, mg)	Yield Ratio
**Ultrasonication (UE)** **(40 kHz ice-bathed, 16 h.)**	200 mg + EtOH, 20 mL	14 mg	7.0%
	200 mg + DIW, 20 mL	9 mg	4.5%
	200 mg + NMP, 20 mL	6 mg	3.0%
**Heating (TE)** **(80 °C, 16 h.)**	200 mg + EtOH, 20 mL	30 mg	15.0%
	200 mg + DIW, 20 mL	4 mg	2.0%
	200 mg + NMP, 20 mL	3 mg	1.5%

**Table 3:** The zeta potential of MoS_2_ nanoparticles synthesized using different methods and solvents.

	Ethanol (EtOH)	Deionized Water (DIW)	N-Methyl-2-Pyrrolidone (NMP)
**Ultrasonication (UE)** **(40 kHz ice-bathed, 16 h.)**	−28.43 ± 4.15 mV	−8.58 ± 2.92 mV	26.13 ± 3.21 mV
**Heating (TE)** **(80 °C, 16 h.)**	−24.27 ± 5.26 mV	−4.88 ± 0.06 mV	41.57 ± 4.61 mV

**Table 4:** Performance evaluation of MoS_2_ nanoparticles.

Synthesis Method	Solvent/Precursors	Yield (%)	Average Particle Size (nm)	PL Quantum Yield (%)	Photothermal Performance (Power Conversion Efficiency (PCE) or ΔT)	Reference
Thermal Exfoliation (TE)	Ethanol/MoS_2_ Powder	15%	~4.1	1.55%	ΔT ≈ 28.4 °C at 300 s, 1.0 W/cm^2^; PCE = 25.86%	This Work
Ultrasonic Exfoliation (UE)	Ethanol/MoS_2_ Powder	7%	~4.0	2.55%	ΔT ≈ 28.1 °C at 300 s, 1.0 W/cm^2^	This Work
Chemical–Ultrasonic Exfoliation (40 kHz)	NaCl, Na_2_CO_3_, LiOH/MoS_2_ Powder		1.5–7	4.84%		[36]
Hydrothermal Synthesis	Water + HCl/(NH_4_)_2_MoS_4_ + GSH		4.4	20.4%		[91]
Chemical–Hydrothermal Exfoliation (Na intercalation)	Water/MoS_2_ Powder + Na sand	11%	3.5	11%		[92]
Cryo-mediated exfoliation	Liquid Nitrogen/MoS_2_ Powder	1%	2.5	10%		[93]
Nebulization–Condensation Method	Lithium Niobate/MoS_2_ Powder	9.65%	8.25	10.7%		[94]
Solvent–Thermal Method	1-Octadecene/(NH_4_)_2_MoS_4,_ Oleic Acid, Oleylamine		4.5 nm		ΔT ≈ 10.4 °C at 300 s, 1 W/cm^2^; PCE = 13.18%	[95]
Ultrasonic–Thermal Exfoliation (UE)	NMP/MoS_2_ Powder		5.3 nm		ΔT ≈ 25 °C at 300 s, 1.5 W/cm^2^	[96]

## Data Availability

The original contributions presented in this study are included in the article/Supplementary Material. Further inquiries can be directed to the corresponding authors.

## Supplementary Materials

The following supporting information can be downloaded at: https://www.mdpi.com/article/10.3390/nano16171058/s1, Finite element analysis for the photothermal test: Figure S1. The geometry of the finite element model; Figure S2. The numerical algorithm for determining the heat source s(t) by a piecewise continuous function. ε is a preset small positive number; Figure S3. The numerical fitting results by the finite element analysis using piecewise continuous functions to approximate the heat source s(t). The calculated temperature is assumed to be at T(r=0, t) and compared with the experimental measurement. The simulation case is for MoS_2_ nanoparticles exfoliated by ultrasound in ethanol; Figure S4. Temperature distribution from numerical analysis by the finite element analysis using the 3rd piecewise continuous functions in Figure S3. The case is for MoS_2_ nanoparticles exfoliated by ultrasound in ethanol. Table S1. Thermal Properties of Water for Finite Element Analysis; Fourier Transform Infrared (FTIR) spectroscopy [97,98,99,100,101,102,103,104,105]: Figure S5. FTIR of MoS2 nanoparticles synthesized using different methods and solvents; Table S2. Vibrational modes of major FTIR peaks.

## References

[1] Mitchell P.C.H., Outteridge T., Kloska K., McMahon S., Epshteyn Y., Sebenik R.F., Burkin A.R., Dorfler R.R., Laferty J.M., Leichtfried G. (2020). Molybdenum and Molybdenum Compounds. Ullmann’s Encyclopedia of Industrial Chemistry.

[2] Srivastava M., Banerjee S., Bairagi S., Singh P., Kumar B., Singh P., Kale R.D., Mulvihill D.M., Ali S.W. (2024). Recent Progress in Molybdenum Disulfide (MoS_2_) Based Flexible Nanogenerators: An Inclusive Review. Chem. Eng. J..

[3] Splendiani A., Sun L., Zhang Y., Li T., Kim J., Chim C.Y., Galli G., Wang F. (2010). Emerging Photoluminescence in Monolayer MoS_2_. Nano Lett..

[4] Wu F.Y., Cheng Y.S., Wang D.M., Li M.L., Lu W.S., Xu X.Y., Zhou X.H., Wei X.W. (2020). Nitrogen-Doped MoS_2_ Quantum Dots: Facile Synthesis and Application for the Assay of Hematin in Human Blood. Mater. Sci. Eng. C.

[5] Thomas N., Mathew S., Nair K.M., O’Dowd K., Forouzandeh P., Goswami A., McGranaghan G., Pillai S.C. (2021). 2D MoS_2_: Structure, Mechanisms, and Photocatalytic Applications. Mater. Today Sustain..

[6] Obodo P.C., Obodo K.O., Aigbe U.O., Aigbodion V. (2025). Recent Advances in Molybdenum Disulfide (MoS_2_) and MXene-based Heterostructures for Photovoltaic and Water Splitting Applications: A Review. ChemistrySelect.

[7] Zhang L., Wang N., Li Y. (2023). Design, Synthesis, and Application of Some Two-Dimensional Materials. Chem. Sci..

[8] Fang C., Wu X., Yang F., Qiao R. (2018). Flow of Quasi-Two Dimensional Water in Graphene Channels. J. Chem. Phys..

[9] Komsa H.-P., Krasheninnikov A.V. (2012). Effects of Confinement and Environment on the Electronic Structure and Exciton Binding Energy of MoS_2_ from First Principles. Phys. Rev. B.

[10] Gan Z.X., Liu L.Z., Wu H.Y., Hao Y.L., Shan Y., Wu X.L., Chu P.K. (2015). Quantum Confinement Effects across Two-Dimensional Planes in MoS_2_ Quantum Dots. Appl. Phys. Lett..

[11] Kuc A., Zibouche N., Heine T. (2011). Influence of Quantum Confinement on the Electronic Structure of the Transition Metal Sulfide *T*S_2_. Phys. Rev. B.

[12] Jiang J., Chen Z., Hu Y., Xiang Y., Zhang L., Wang Y., Wang G.C., Shi J. (2021). Flexo-Photovoltaic Effect in MoS_2_. Nat. Nanotechnol..

[13] Guo Y., Li J. (2020). MoS_2_ Quantum Dots: Synthesis, Properties and Biological Applications. Mater. Sci. Eng. C.

[14] Sinha S.S., Yadgarov L., Aliev S.B., Feldman Y., Pinkas I., Chithaiah P., Ghosh S., Idelevich A., Zak A., Tenne R. (2021). MoS_2_ and WS_2_ Nanotubes: Synthesis, Structural Elucidation, and Optical Characterization. J. Phys. Chem. C.

[15] García de Arquer F.P., Talapin D.V., Klimov V.I., Arakawa Y., Bayer M., Sargent E.H. (2021). Semiconductor Quantum Dots: Technological Progress and Future Challenges. Science.

[16] Lims S.C., Tran N.A., Dao V.-D., Pham P.V. (2025). The World of Quantum Dot-Shaped Nanoparticles: Nobel Prize in Chemistry 2023: Advancements and Prospectives. Coord. Chem. Rev..

[17] Chen L., Chuang Y., Chen C.-W., Dong C.-D. (2022). Facile Synthesis of MoS_2_/ZnO Quantum Dots for Enhanced Visible-Light Photocatalytic Performance and Antibacterial Applications. Nano-Struct. Nano-Objects.

[18] Chen X., Park Y.J., Kang M., Kang S.K., Koo J., Shinde S.M., Shin J., Jeon S., Park G., Yan Y. (2018). CVD-Grown Monolayer MoS_2_ in Bioabsorbable Electronics and Biosensors. Nat. Commun..

[19] Neacșa A., Ramadan I.N., Diniță A., Iacob Ș.V., Ilincă C.N., Laudacescu E.V. (2025). Can Non-Phase-Transformation Heat Treatments Improve the Strength Properties of Materials?. Materials.

[20] Said Z., Pandey A.K., Tiwari A.K., Kalidasan B., Jamil F., Thakur A.K., Tyagi V.V., Sarı A., Ali H.M. (2024). Nano-Enhanced Phase Change Materials: Fundamentals and Applications. Prog. Energy Combust. Sci..

[21] Hu L., Zhao Q., Huang S., Zheng J., Guan X., Patterson R., Kim J., Shi L., Lin C.-H., Lei Q. (2021). Flexible and Efficient Perovskite Quantum Dot Solar Cells via Hybrid Interfacial Architecture. Nat. Commun..

[22] Shilpa G., Kumar P.M., Kumar D.K., Deepthi P.R., Sadhu V., Sukhdev A., Kakarla R.R. (2023). Recent Advances in the Development of High Efficiency Quantum Dot Sensitized Solar Cells (QDSSCs): A Review. Mater. Sci. Energy Technol..

[23] Murphy C.J. (2002). Peer Reviewed: Optical Sensing with Quantum Dots. Anal. Chem..

[24] Lesiak A., Drzozga K., Cabaj J., Bański M., Malecha K., Podhorodecki A. (2019). Optical Sensors Based on II-VI Quantum Dots. Nanomaterials.

[25] Engström O., Kaniewska M. (2008). Deep Level Transient Spectroscopy in Quantum Dot Characterization. Nanoscale Res. Lett..

[26] Oksanen J., Prunnila M. (2023). Thermal Management in Quantum-Dot LEDs through Optical Thermodynamics. Joule.

[27] Yan Y., Gong J., Chen J., Zeng Z., Huang W., Pu K., Liu J., Chen P. (2019). Recent Advances on Graphene Quantum Dots: From Chemistry and Physics to Applications. Adv. Mater..

[28] Zare I., Zahed Nasab S., Rahi A., Ghaee A., Koohkhezri M., Ramezani Farani M., Madadi Gholipour H., Atabaki A.H., Hamblin M.R., Mostafavi E. (2025). Antibacterial Carbon Materials-Based Quantum Dots: From Synthesis Strategies to Antibacterial Properties for Diagnostic and Therapeutic Applications in Wound Healing. Coord. Chem. Rev..

[29] Dong X., Liang W., Meziani M.J., Sun Y.-P., Yang L. (2020). Carbon Dots as Potent Antibacterial Agents. Theranostics.

[30] Seth S., Karthikeyan, Rathinasabapathi P., Selvarajan E., Samuel M.S., Chandrasekar N., Balaji R. (2023). Quantum Dots as Antibacterial Agents. Carbon and Graphene Quantum Dots for Biomedical Applications.

[31] Montazer L., Mahani M., Khakbaz F., Divsar F., Yoosefian M. (2024). Carbon Quantum Dots and Gold Nanostructures on Photothermal Therapy for Cancer Treatment. J. Photochem. Photobiol. A Chem..

[32] Dar M.S., Tabish T.A., Thorat N.D., Swati G., Sahu N.K. (2023). Photothermal Therapy Using Graphene Quantum Dots. APL Bioeng..

[33] Pareek A., Kumar D., Pareek A., Gupta M.M. (2025). Advancing Cancer Therapy with Quantum Dots and Other Nanostructures: A Review of Drug Delivery Innovations, Applications, and Challenges. Cancers.

[34] Li B., Jiang L., Li X., Ran P., Zuo P., Wang A., Qu L., Zhao Y., Cheng Z., Lu Y. (2017). Preparation of Monolayer MoS_2_ Quantum Dots Using Temporally Shaped Femtosecond Laser Ablation of Bulk MoS_2_ Targets in Water. Sci. Rep..

[35] Liu Q., Hu C., Wang X. (2016). A Facile One-Step Method to Produce MoS_2_ Quantum Dots as Promising Bio-Imaging Materials. RSC Adv..

[36] Wu J.-Y., Zhang X.-Y., Ma X.-D., Qiu Y.-P., Zhang T. (2015). High Quantum-Yield Luminescent MoS_2_ Quantum Dots with Variable Light Emission Created via Direct Ultrasonic Exfoliation of MoS_2_ Nanosheets. RSC Adv..

[37] Agarwal K., Rai H., Mondal S. (2023). Quantum Dots: An Overview of Synthesis, Properties, and Applications. Mater. Res. Express.

[38] Jalali H.B., Sadeghi S., Dogru Yuksel I.B., Onal A., Nizamoglu S. (2022). Past, Present and Future of Indium Phosphide Quantum Dots. Nano Res..

[39] Chen Z., Zhao C., Zhou X., Xiao L., Li Z., Zhang Y. (2023). A Review of Top-Down Strategies for the Production of Quantum-Sized Materials. Small Sci..

[40] Chatterjee S., Maitra U. (2017). In Situ Formation of Luminescent CdSe QDs in a Metallohydrogel: A Strategy towards Synthesis, Isolation, Storage and Re-Dispersion of the QDs. Nanoscale.

[41] Guo Y., Zhang L., Zhang S., Yang Y., Chen X., Zhang M. (2015). Fluorescent Carbon Nanoparticles for the Fluorescent Detection of Metal Ions. Biosens. Bioelectron..

[42] Häckl K., Kunz W. (2018). Some Aspects of Green Solvents. C. R. Chim..

[43] Winterton N. (2021). The Green Solvent: A Critical Perspective. Clean Technol. Environ. Policy.

[44] Cvjetko Bubalo M., Vidović S., Radojčić Redovniković I., Jokić S. (2015). Green Solvents for Green Technologies. Clean Technol. Environ. Policy.

[45] Sim D.M., Han H.J., Yim S., Choi M.-J., Jeon J., Jung Y.S. (2017). Long-Term Stable 2H-MoS_2_ Dispersion: Critical Role of Solvent for Simultaneous Phase Restoration and Surface Functionalization of Liquid-Exfoliated MoS_2_. ACS Omega.

[46] Kaushik S., Nemala S.S., Kumar M., Negi D., Dhal B., Saini L., Banavath R., Saha S., Sharma S., Kalon G. (2022). High-Yield Exfoliation of MoS_2_ Nanosheets by a Novel Spray Technique and the Importance of Soaking and Surfactants. Nano-Struct. Nano-Objects.

[47] O’Neill A., Khan U., Coleman J.N. (2012). Preparation of High Concentration Dispersions of Exfoliated MoS_2_ with Increased Flake Size. Chem. Mater..

[48] Wang H., Chen B., Zhang X., Liu S., Zhu B., Wang J., Wu K., Chen J. (2015). Ethanol Catalytic Deposition of MoS_2_ on Tapered Fiber. Photonics Res..

[49] Huang H., Liu N., Wang X., Zhong M., Huang X. (2022). Application of Hydrothermal and Solvothermal Method in Synthesis of MoS_2_. Rev. Chim..

[50] Li Z., Fan R., Hu Z., Li W., Zhou H., Kang S., Zhang Y., Zhang H., Wang G. (2020). Ethanol Introduced Synthesis of Ultrastable 1T-MoS_2_ for Removal of Cr(VI). J. Hazard. Mater..

[51] Varrla E., Backes C., Paton K.R., Harvey A., Gholamvand Z., McCauley J., Coleman J.N. (2015). Large-Scale Production of Size-Controlled MoS_2_ Nanosheets by Shear Exfoliation. Chem. Mater..

[52] Smith R.J., King P.J., Lotya M., Wirtz C., Khan U., De S., O’Neill A., Duesberg G.S., Grunlan J.C., Moriarty G. (2011). Large-Scale Exfoliation of Inorganic Layered Compounds in Aqueous Surfactant Solutions. Adv. Mater..

[53] Yao Y., Lin Z., Li Z., Song X., Moon K.-S., Wong C. (2012). Large-Scale Production of Two-Dimensional Nanosheets. J. Mater. Chem..

[54] Museux N., Perez L., Autrique L., Agay D. (2012). Skin Burns after Laser Exposure: Histological Analysis and Predictive Simulation. Burns.

[55] Svobodova B., Kloudova A., Ruzicka J., Kajtmanova L., Navratil L., Sedlacek R., Suchy T., Jhanwar-Uniyal M., Jendelova P., Machova Urdzikova L. (2019). The Effect of 808 Nm and 905 Nm Wavelength Light on Recovery after Spinal Cord Injury. Sci. Rep..

[56] Mitchell U.H., Myrer J.W., Johnson A.W., Hilton S.C. (2011). Restless Legs Syndrome and Near-Infrared Light: An Alternative Treatment Option. Physiother. Theory Pract..

[57] Rojas J.C., Gonzalez-Lima F. (2011). Low-Level Light Therapy of the Eye and Brain. Eye Brain.

[58] Zhang R., Qu J. (2023). The Mechanisms and Efficacy of Photobiomodulation Therapy for Arthritis: A Comprehensive Review. Int. J. Mol. Sci..

[59] Dompe C., Moncrieff L., Matys J., Grzech-Leśniak K., Kocherova I., Bryja A., Bruska M., Dominiak M., Mozdziak P., Skiba T. (2020). Photobiomodulation—Underlying Mechanism and Clinical Applications. J. Clin. Med..

[60] Ruokolainen J., Malinen M., Råback P., Zwinger T., Takala E., Kataja J., Gillet-Chaulet F., Ilvonen S., Gladstone R., Byckling M. (2023). ElmerCSC/Elmerfem: Elmer 9.0.

[61] Liu Y., Zhong Q., Chen K., Zhou J., Yang X., Chen W. (2017). Morphologies Controllable Synthesis of MoS_2_ by Hot-Injection Method: From Quantum Dots to Nanosheets. J. Mater. Sci. Mater. Electron..

[62] Kariachan S., Shibu J., Velayudhan P., Sidharthan S.K., Velayudhan P., Simon S.M., Kasai H., Okubo K., Thomas S., Oka K. (2026). Exploring the Advances in 2D Materials as a Quest for Energy Storage Electrode Materials. RSC Adv..

[63] Yi S., Babadagli T., Li H. (2020). Stabilization of Nickel Nanoparticle Suspensions with the Aid of Polymer and Surfactant: Static Bottle Tests and Dynamic Micromodel Flow Tests. Pet. Sci..

[64] Chikan V., Kelley D.F. (2002). Size-Dependent Spectroscopy of MoS_2_ Nanoclusters. J. Phys. Chem. B.

[65] Song C., Wang Z., Yin Z., Xiao D., Ma D. (2022). Principles and Applications of Photothermal Catalysis. Chem Catal..

[66] Fu Y., Liang F., He C., Yu H., Zhang H., Chen Y.-F. (2023). Photon-Phonon Collaboratively Pumped Laser. Nat. Commun..

[67] Cao H., Wang H., Huang Y., Sun Y., Shi S., Tang M. (2017). Quantification of Gold(III) in Solution and with a Test Stripe via the Quenching of the Fluorescence of Molybdenum Disulfide Quantum Dots. Microchim. Acta.

[68] Ryou J., Kim Y.-S., KC S., Cho K. (2016). Monolayer MoS_2_ Bandgap Modulation by Dielectric Environments and Tunable Bandgap Transistors. Sci. Rep..

[69] Kobayashi K., Yamauchi J. (1995). Electronic Structure and Scanning-Tunneling-Microscopy Image of Molybdenum Dichalcogenide Surfaces. Phys. Rev. B.

[70] Yun W.S., Han S.W., Hong S.C., Kim I.G., Lee J.D. (2012). Thickness and Strain Effects on Electronic Structures of Transition Metal Dichalcogenides: 2H-*M**X*_2_ Semiconductors (*M* = Mo, W; *X* = S, Se, Te). Phys. Rev. B.

[71] Cheiwchanchamnangij T., Lambrecht W.R.L. (2012). Quasiparticle Band Structure Calculation of Monolayer, Bilayer, and Bulk MoS_2_. Phys. Rev. B.

[72] Qiu D.Y., da Jornada F.H., Louie S.G. (2013). Optical Spectrum of MoS_2_: Many-Body Effects and Diversity of Exciton States. Phys. Rev. Lett..

[73] Gupta A., Arunachalam V., Vasudevan S. (2016). Liquid-Phase Exfoliation of MoS_2_ Nanosheets: The Critical Role of Trace Water. J. Phys. Chem. Lett..

[74] Chen P.-C., Lin C.-P., Hong C.-J., Yang C.-H., Lin Y.-Y., Li M.-Y., Li L.-J., Yu T.-Y., Su C.-J., Li K.-S. (2019). Effective N-Methyl-2-Pyrrolidone Wet Cleaning for Fabricating High-Performance Monolayer MoS_2_ Transistors. Nano Res..

[75] Kira M., Jahnke F., Koch S.W. (1999). Quantum Theory of Secondary Emission in Optically Excited Semiconductor Quantum Wells. Phys. Rev. Lett..

[76] Faraji M., Yamini Y., Salehi N. (2021). Characterization of Magnetic Nanomaterials. Magnetic Nanomaterials in Analytical Chemistry.

[77] Wang Y., Dou W. (2024). Interband and Intraband Transitions, as Well as Charge Mobility in Driven Two-Band Model with Electron Phonon Coupling. J. Chem. Phys..

[78] Vlasenko N.A. (2005). Momentum Relaxation of Hot Electrons during Radiative Intraband Indirect Transitions in ZnS:Cr. Semicond. Phys. Quantum Electron. Optoelectron..

[79] Hari Krishna P., Ramrakhiani M. (2010). Nano Particle Size Effect on Photo-Luminescence. Int. J. Nanotechnol. Appl..

[80] Wu X., Suo Y., Shi H., Liu R., Wu F., Wang T., Ma L., Liu H., Cheng Z. (2020). Deep-Tissue Photothermal Therapy Using Laser Illumination at NIR-IIa Window. Nano-Micro Lett..

[81] Jiang Q., Zeng W., Zhang C., Meng Z., Wu J., Zhu Q., Wu D., Zhu H. (2017). Broadband Absorption and Enhanced Photothermal Conversion Property of Octopod-like Ag@Ag_2_S Core@shell Structures with Gradually Varying Shell Thickness. Sci. Rep..

[82] Marinov A.D., Clancy A.J., Howard C.A., Cullen P.L. (2026). XPS Peak-Fitting of 2H MoS_2_, 1T MoS_2_, and MoS_2_-X Nanosheets in MoS_2_ Powders and Battery Electrodes After Ar^+^ Ion Depth-Profiling. ACS Appl. Nano Mater..

[83] Munusami V., Arutselvan K., Vadivel S., Govindasamy S. (2022). High Sensitivity LPG and H_2_ Gas Sensing Behavior of MoS_2_/Graphene Hybrid Sensors Prepared by Facile Hydrothermal Method. Ceram. Int..

[84] Bao Y., Yang M., Tan S.J.R., Liu Y.P., Xu H., Liu W., Nai C.T., Feng Y.P., Lu J., Loh K.P. (2016). Substoichiometric Molybdenum Sulfide Phases with Catalytically Active Basal Planes. J. Am. Chem. Soc..

[85] Menzel J.P., Noble B.B., Blinco J.P., Barner-Kowollik C. (2021). Predicting Wavelength-Dependent Photochemical Reactivity and Selectivity. Nat. Commun..

[86] Drozd G.T., Weltzin T., Skiffington S., Lee D., Valiev R., Kurtén T., Madison L.R., He Y., Gargano L. (2024). Wavelength-Resolved Quantum Yields for Vanillin Photochemistry: Self-Reaction and Ionic-Strength Implications for Wildfire Brown Carbon Lifetime. Environ. Sci. Atmos..

[87] Maafi M. (2024). On Photokinetics under Polychromatic Light. Front. Chem..

[88] Porrès L., Holland A., Pålsson L.O., Monkman A.P., Kemp C., Beeby A. (2006). Absolute Measurements of Photoluminescence Quantum Yields of Solutions Using an Integrating Sphere. J. Fluoresc..

[89] Greenham N.C., Samuel I.D.W., Hayes G.R., Phillips R.T., Kessener Y.A.R.R., Moratti S.C., Holmes A.B., Friend R.H. (1995). Measurement of Absolute Photoluminescence Quantum Efficiencies in Conjugated Polymers. Chem. Phys. Lett..

[90] De Mello J.C., Wittmann H.F., Friend R.H. (1997). An Improved Experimental Determination of External Photoluminescence Quantum Efficiency. Adv. Mater..

[91] Xie L., Yang Y., Gong G., Feng S., Liu D. (2022). One-Step Hydrothermal Synthesis of Highly Fluorescent MoS_2_ Quantum Dots for Lead Ion Detection in Aqueous Solutions. Nanomaterials.

[92] Zhou K., Zhang Y., Xia Z., Wei W. (2016). As-Prepared MoS_2_ Quantum Dot as a Facile Fluorescent Probe for Long-Term Tracing of Live Cells. Nanotechnology.

[93] Wang Y., Liu Y., Zhang J., Wu J., Xu H., Wen X., Zhang X., Tiwary C.S., Yang W., Vajtai R. (2017). Cryo-Mediated Exfoliation and Fracturing of Layered Materials into 2D Quantum Dots. Sci. Adv..

[94] Marqus S., Ahmed H., Ahmed M., Xu C., Rezk A.R., Yeo L.Y. (2018). Increasing Exfoliation Yield in the Synthesis of MoS_2_ Quantum Dots for Optoelectronic and Other Applications through a Continuous Multicycle Acoustomicrofluidic Approach. ACS Appl. Nano Mater..

[95] Liu J., Cui E., Zhang Q., Xie D. (2024). MoS_2_-Based Nanocomposites with High Photothermal Conversion Efficiency for Combinational Photothermal/Photodynamic Tumor Therapy. J. Alloys Compd..

[96] Wang J., Tan X., Pang X., Liu L., Tan F., Li N. (2016). MoS_2_ Quantum Dot@Polyaniline Inorganic–Organic Nanohybrids for In Vivo Dual-Modal Imaging Guided Synergistic Photothermal/Radiation Therapy. ACS Appl. Mater. Interfaces.

[97] Ghasemipour P., Fattahi M., Rasekh B., Yazdian F. (2020). Developing the Ternary ZnO Doped MoS_2_ Nanostructures Grafted on CNT and Reduced Graphene Oxide (RGO) for Photocatalytic Degradation of Aniline. Sci. Rep..

[98] Yang Y., Yu Y., Cui C., Liu X., Li Y., Liu P., Hui F. (2026). Interfacial Engineering of MoS_2_ Thin Films for Wettability-Dependent Resistive Switching and Neuromorphic Behaviors. Nanomaterials.

[99] Shaltout A.A., Mostafa N.Y., Mahani R.M., Ahmed S.I., Allam M.A., Alzahrani E., Wahba H.H. (2020). Investigation of structural and optical properties of molybdenum disulfide flakes/polyvinylidene fluoride nanocomposites. J. Mater. Res. Technol..

[100] Nadeem M.T., Aqeel R., Zafar A., Nisar A., Khan M.I., Ahmad M. (2025). Synergic effect of a MoS_2_–V_2_O_5_ heterostructure as an advanced catalyst for photocatalytic degradation of methylene blue. New J. Chem..

[101] Rodríguez-Ocanto N., González-Gómez W.S., Rodríguez-Gattorno G., Padron-Hernandez W., Ruiz-Gómez M.A. (2025). Sustainable colloidal ink for inkjet printing of 1T/2H-MoS_2_ based photodetectors on flexible substrate. Mater. Sci. Semicond. Process..

[102] Zhao J., Zhang Z., Yang S., Zheng H., Li Y. (2013). Facile synthesis of MoS_2_ nanosheet-silver nanoparticles composite for surface enhanced Raman scattering and electrochemical activity. J. Alloys Compd..

[103] Liu B., Ma Y., Zhang A., Chen L., Abbas A.N., Liu Y., Shen C., Wan H., Zhou C. (2016). High-Performance WSe_2_ Field-Effect Transistors via Controlled Formation of In-Plane Heterojunctions. ACS Nano.

[104] Lalithambika K.C., Shanmugapriya K., Sriram S. (2019). Photocatalytic activity of MoS_2_ nanoparticles: An experimental and DFT analysis. Appl. Phys. A.

[105] Yi M., Zhang C. (2018). The synthesis of two-dimensional MoS_2_ nanosheets with enhanced tribological properties as oil additives. RSC Adv..

