# Supplementary material for: Comparison Between Ultrasonic and Thermal Exfoliation for Molybdenum Disulfide Nanoparticles—A Multifunctional Study
Source: Nanomaterials (Basel). 2026 Aug 25;16(17):1058. doi: 10.3390/nano16171058 (PMC13567494; doi:10.3390/nano16171058)
Supplement: Supplementary file 1 [file nanomaterials-16-01058-s001.zip › 2026 0806 Supplementary MDPI nanomaterials 1.pdf]

Supplementary Material S1

1 Finite Element Model

The geometric model of finite element analysis is shown in Figure S1, where an octant-cylinder of the sample is set up to represent the suspension of MoS_2 nanoparticles in a scintillation vial (12.25 mm in diameter). A laser beam with a spot diameter of 2 mm irradiates through the cylinder horizontally. Note that only the photothermal test for samples of MoS_2 nanoparticles synthesized by ultrasonication in ethanol is simulated due to their significant temperature rise among all samples in the photothermal test.

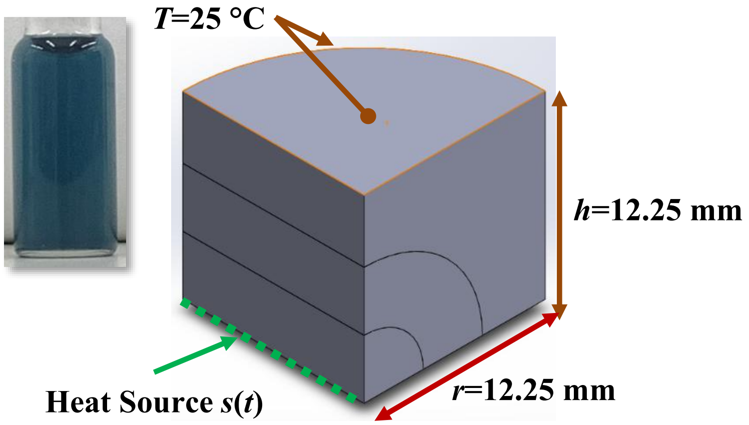

Figure S1. The geometry of the finite element model.

Several assumptions are made for the analysis. (1) Assuming a hydrostatic condition of water during testing, and thus, only heat conduction governs the heat transfer. This assumption is judged from experiments that temperature increases are not too high (< 55°C) to cause significant convection; (2) assuming the MoS_2 nanoparticles suspension is dilute and homogeneous; therefore, water represents the simulation domain. (3) The power from the laser beam (1 W) continuously irradiates the sample without energy loss. This assumption is based on the fact that almost no reflection, scattering, or absorption of the laser beam occurred, and the scintillation vial maintained room temperature throughout the test.

1.1 Governing Equation

Note that the governing equation based on previous assumptions is heat conduction only:

$$k\nabla^2 T(\boldsymbol{r},t) + s(\boldsymbol{r},t) = \rho c_p \frac{\partial T(\boldsymbol{r},t)}{\partial t} \qquad \text{(S1)}$$

Where $\nabla^2 \equiv (\frac{\partial^2}{\partial r^2} + \frac{1}{r}\frac{\partial}{\partial r} + \frac{1}{r^2}\frac{\partial^2}{\partial \theta^2} + \frac{\partial^2}{\partial z^2})$ is the Laplacian operator in cylindrical coordinates; k, ρ, c_p are thermal conductivity, density, and isobaric specific heat, respectively; $s(r,t)$ is the heat source (W/m^3). The source is unknown, generated from continuous irradiation by an 808-nm laser with a power of 1 W. It needs to be determined from temperature measures in the photothermal tests. Since the assumption is that a homogeneous suspension of MoS_2 nanoparticles , the source term can be approximated as

$$s(\boldsymbol{r}, t) \approx s(t) \tag{S2}$$

1.2 Boundary and Initial Conditions

The boundary condition is a constant temperature of 25°C on the surface of the domain, to mimic the room temperature when the scintillation vial is being tested. The heat source is assigned to the centerline of the quadrant cylinder. This approximation is due to the much smaller radius of the laser beam, which is only about 1/10 of the scintillation vial radius.

The initial condition is obtained from the experimental data at t=0 throughout the domain. This is the state of the samples before the photothermal test.

1.3 Material Properties

Thermal properties of water at various temperatures are listed in Table S1. Since the variation (standard deviation) of these properties is small throughout the photothermal test, it is assumed that these properties are constant using their average values for the finite element analysis.

Table S1. Thermal Properties of Water for Finite Element Analysis.

Temperature (°C)	*Density ρ (kg/m^3)	**Thermal conductivity k (W/m·K)	***Isobaric Specific Heat c_p (J/g °C)
20	0.999700	0.598030	4.184400
30	0.998207	0.614500	4.181600
40	0.995649	0.628560	4.180100
50	0.992215	0.640600	4.179600
60	0.988040	0.650910	4.181500
Average	0.994762	0.626520	4.181440
Std. deviation	0.004709	0.020937	0.001869

(Source: *https://www.engineeringtoolbox.com/water-density-specific-weight-d_595.html,
**https://www.engineeringtoolbox.com/water-liquid-gas-thermal-conductivity-temperature-pressure-d_2012.html,
***https://www.engineeringtoolbox.com/specific-heat-capacity-water-d_660.html)

2 Optimal Solution and Numerical Algorithm

The unknown heat source $s(t)$ can be estimated by minimizing the differences in temperatures from the experiment and the finite element solution $T(r=0, t)$. This minimization problem is stated as

$$\min_{s} \sqrt{\sum_{i=0}^{n} \frac{\left(T^{expriment}(t_i) - T^{Finite\ Element}(r=0,t_i)\right)^2}{n}} \tag{S3}$$

In other words, an optimal value of $s(t)$ is determined by minimizing the root mean square error (RMSE) between the analysis and the experiment.

However, since the heat source $s(t)$ is an unknown function of time, the function cannot be estimated blindly. The following algorithm is constructed for a systematic modification from an initial guess of $s(t)$.

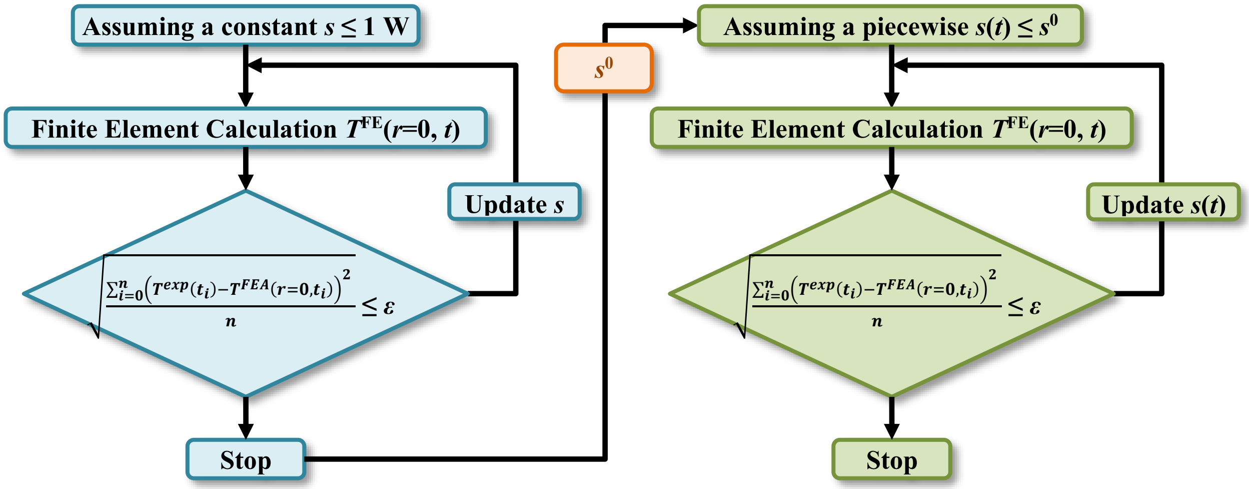

Figure S2. The numerical algorithm for determining the heat source *s*(*t*) by a piecewise continuous function. *ε* is a preset small positive number.

Note that a *piecewise continuous* function is used to approximate $s(t)$. The time intervals of the piecewise continuous function can be arbitrarily determined, but it is strongly recommended to follow the experimental data. All values of this piecewise continuous function should be less than s^0 from the preliminary round of optimization on the left side.

3 Results

Using the case of MoS_2 nanoparticles exfoliated by ultrasound in ethanol as an example, the finite element analysis is carried out by SOLIDWORKS Simulation®. Following the proposed numerical algorithm, the optimal constant heat source is determined to be $s_0 \approx 0.040625$ W to minimize the RMSE between experimental and simulation data at $T(r=0, t)$. Then, several different piecewise continuous functions were further tested to minimize RMSE between the two datasets.

Figure S3 shows the results of 3 different test functions for $s(t)$ with the experimental data. The 1st one is a preliminary constant s estimated from the 1st round of optimization. The 3rd function, as shown in the inset, closely matches the experimental measures, is a stepwise piecewise continuous function at various time intervals. This numerical analysis demonstrates the feasibility of the proposed algorithm for the determination of the heat source with a stable convergence to the experimental data.

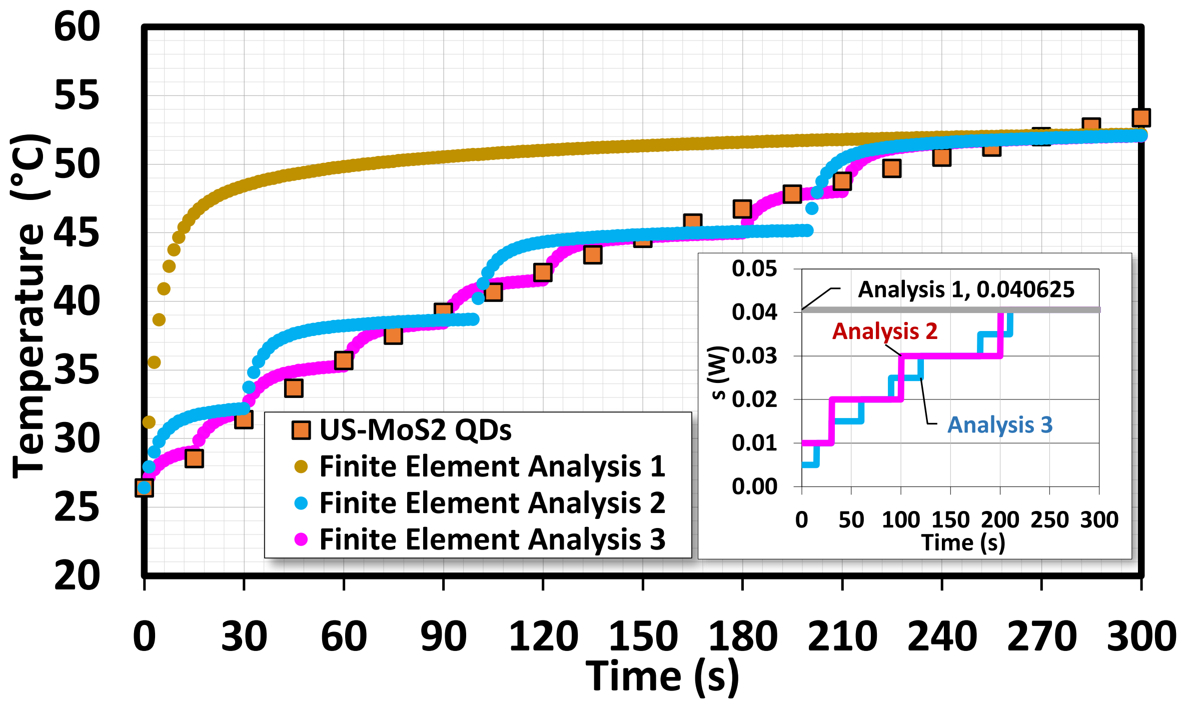

Figure S3. The numerical fitting results by the finite element analysis using piecewise continuous functions to approximate the heat source *s*(*t*). The calculated temperature is assumed to be at *T*(*r*=0, *t*) and compared with the experimental measurement. The simulation case is for MoS_2 nanoparticles exfoliated by ultrasound in ethanol.

Figure S4 shows the temperature distribution from finite element analysis using the 3rd function of the heat source. As can be seen in the figure, the heating zone is asymmetrically centered around the line of the laser. This local heating is desirable as we expect the photothermal effect of QDs to be a precision treatment by laser. The heating in a confined, small area implies possible clinical uses.

Other cases show similar convergent results; we skipped these cases for the conciseness of the context.

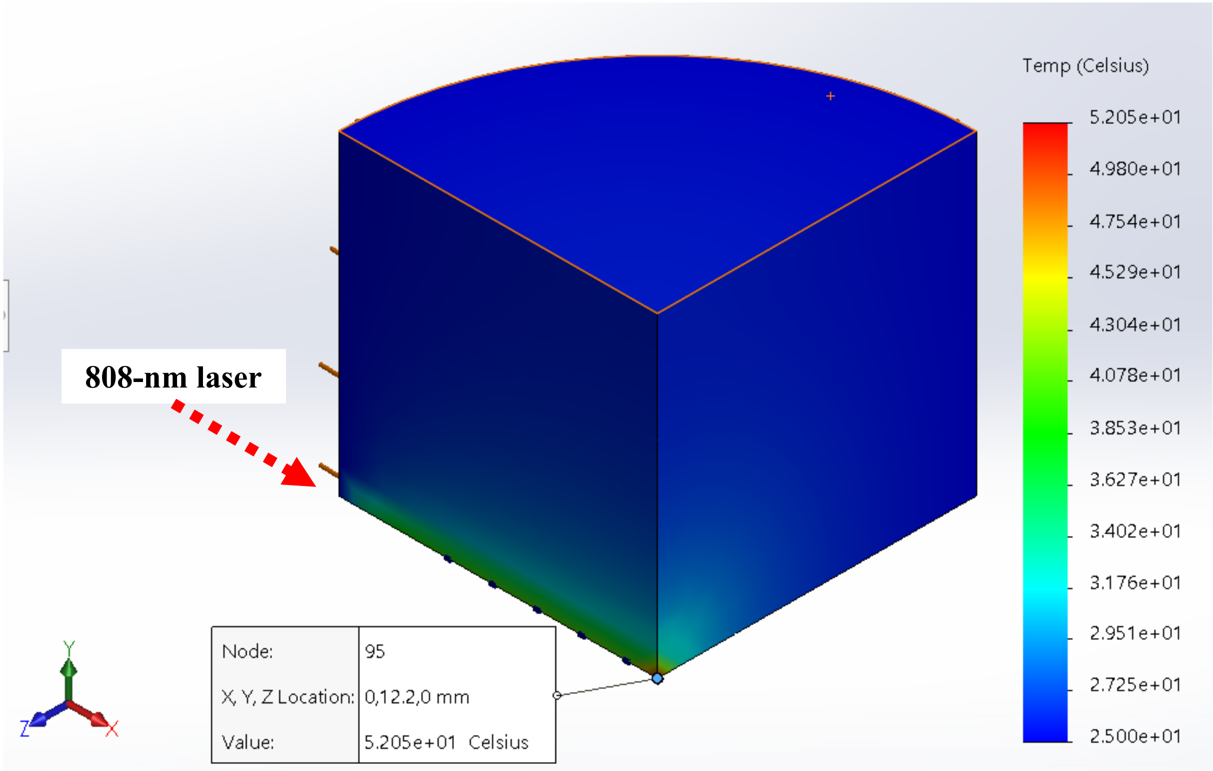

Figure S4. Temperature distribution from numerical analysis by the finite element analysis using the 3rd piecewise continuous functions in Figure S3. The case is for MoS_2 nanoparticles exfoliated by ultrasound in ethanol.

4 Summary

The finite element analysis is briefly presented for the simulation of photothermal effects by MoS_2 nanoparticles in DI water. Using the case of QDs by ultrasonic exfoliation in ethanol as an example, the proposed numerical algorithm is carried out to search for optimal functions of the heat source used in the finite element analysis. A piecewise continuous function of a heat source is tested and shows a good convergence to the experimental data with minimized RMSE. This numerical study shows that the proposed algorithm for an optimal heat source function is stable and practically reliable.
