# Supplementary material for: Comparison Between Ultrasonic and Thermal Exfoliation for Molybdenum Disulfide Nanoparticles—A Multifunctional Study
Source: Nanomaterials (Basel). 2026 Aug 25;16(17):1058. doi: 10.3390/nano16171058 (PMC13567494; doi:10.3390/nano16171058)
Supplement: Supplementary file 1 [file nanomaterials-16-01058-s001.zip › 2026 0806 Supplementary MDPI nanomaterials 2.pdf]

Supplementary Material S2

1 Fourier Transform Infrared (FTIR) Spectroscopy

The FTIR spectra of MoS_2 nanoparticles synthesized using different methods and solvents are shown in Figure S5. Major peaks of the vibrational modes and corresponding molecular structures are marked in the figure and listed in Table S2.

Note that MoS_2 exfoliated in ethanol has two unique peaks. The peak near 803 cm^{-1} is generally assigned to Mo–O stretching vibrations resulting from minor surface oxidation or oxygen coordination (spatial arrangement and chemical bonding of oxygen atoms to Mo). Furthermore, coupled interactions between out-of-plane bending or skeletal vibrations may occur when 2H-MoS_2 is hybridized or functionalized with nearby metallic nanoparticles. The disulfide stretching near 880 cm^{-1} is assigned to localized S-S stretching vibrational modes within sulfur-related defects or some specific polytypic domains.

The broad absorption band in the near-infrared region shown in the UV-Vis-NIR spectra may be due to these defects or surface modification during exfoliation.

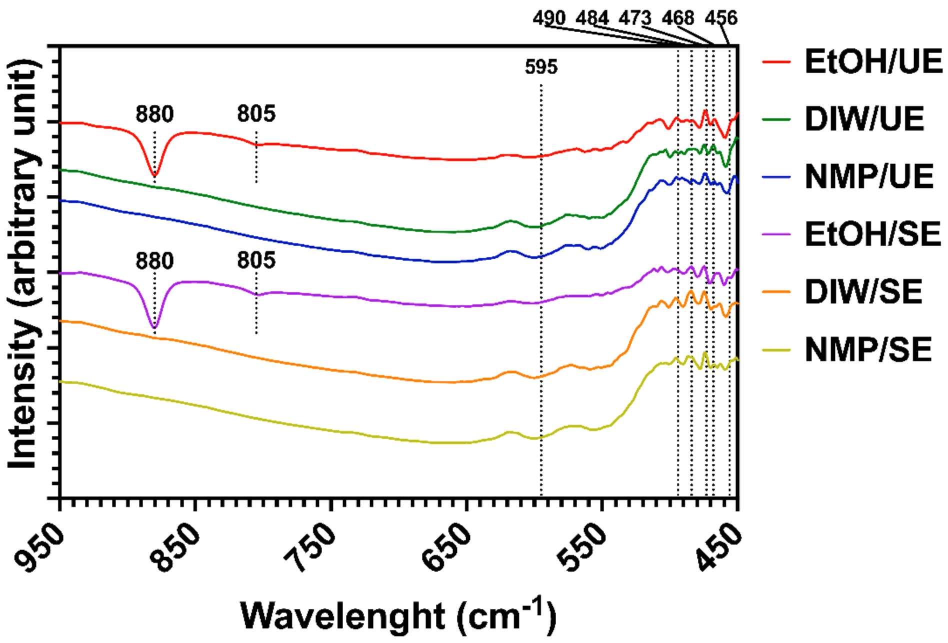

Figure S5. FTIR of MoS_2 nanoparticles synthesized using different methods and solvents.

Table S2. Vibrational modes of major FTIR peaks.

Wavenumber (cm^{-1})	Mode	Reference
**456**	**Mo–S Bond Stretching**	[97-99]
**468**	**Mo–S Bond Stretching shift**	[97-99]
**473**	**Mo–S Bond Stretching shift**	[97-99]
**484**	**Mo–S Bond Stretching shift**	[100,101]
**490 - 494**	**Mo–S Bond Stretching**	[100,101]
**595**	**Mo–S or S-S Stretching**	[102]
**803**	**Asymmetrical stretching or phonon features partial oxidation of Mo**	[103,104]
**879**	**S-S Stretching**	[105]

References

97. Ghasemipour, P., Fattahi, M., Rasekh, B. et al. Developing the Ternary ZnO Doped MoS_2 Nanostructures Grafted on CNT and Reduced Graphene Oxide (RGO) for Photocatalytic Degradation of Aniline. *Sci. Rep.* **2020** 10, 4414. https://doi.org/10.1038/s41598-020-61367-7.
98. Yang, Y.; Yu, Y.; Cui, C.; Liu, X.; Li, Y.; Liu, P.; Hui, F. Interfacial Engineering of MoS_2 Thin Films for Wettability-Dependent Resistive Switching and Neuromorphic Behaviors. *Nanomaterials* **2026**, 16, 959. https://doi.org/10.3390/nano16150959.
99. Shaltout, A. A., Mostafa, N. Y., Mahani, R. M., Ahmed, S. I., Allam, M. A., Alzahrani, E., & Wahba, H. H., Investigation of structural and optical properties of molybdenum disulfide flakes/polyvinylidene fluoride nanocomposites. *J. Mater. Res. Technol.* **2020**, 9(6), 14350-14359. https://doi.org/10.1016/j.jmrt.2020.10.009.
100. Muhammad Tariq Nadeem, Rimsha Aqeel, Amina Zafar, Amjad Nisar, Muhammad Iftikhar Khan, Mashkoor Ahmad; Synergic effect of a MoS_2–V_2O_5 heterostructure as an advanced catalyst for photocatalytic degradation of methylene blue. *New J. Chem.* **2025**; 49 (4): 1171–1181. https://doi.org/10.1039/d4nj04295f.
101. Rodríguez-Ocanto, Nallybe, et al. Sustainable colloidal ink for inkjet printing of 1T/2H-MoS_2 based photodetectors on flexible substrate. *Mater. Sci. Semicond. Process.* **2025**, 200: 110019. https://doi.org/10.1016/j.mssp.2025.110019.
102. Zhao, J., Zhang, Z., Yang, S., Zheng, H., & Li, Y., Facile synthesis of MoS_2 nanosheet-silver nanoparticles composite for surface enhanced Raman scattering and electrochemical activity. *J. Alloys Compd.* **2013**, 559, 87-91. https://doi.org/10.1016/j.jallcom.2013.01.067.
103. Bilu Liu, Yuqiang Ma, Anyi Zhang, Liang Chen, Ahmad N. Abbas, Yihang Liu, Chenfei Shen, Haochuan Wan, Chongwu Zhou; High-Performance WSe_2 Field-Effect Transistors via Controlled Formation of In-Plane Heterojunctions. ACS Nano 24 May 2016; 10 (5): 5153–5160. https://doi.org/10.1021/acsnano.6b00527
104. Lalithambika, K.C., Shanmugapriya, K. & Sriram, S. Photocatalytic activity of MoS_2 nanoparticles: an experimental and DFT analysis. *Appl. Phys.* **2019**. A 125, 817. https://doi.org/10.1007/s00339-019-3120-9.
105. Yi, M., & Zhang, C.. The synthesis of two-dimensional MoS2 nanosheets with enhanced tribological properties as oil additives. *RSC Adv.* **2018**, 8(17), 9564–9573. https://doi.org/10.1039/c7ra12897e.
